# Acidobacteria are active and abundant members of diverse atmospheric H_2_-oxidizing communities detected in temperate soils

**DOI:** 10.1038/s41396-020-00750-8

**Published:** 2020-10-06

**Authors:** Andrew T. Giguere, Stephanie A. Eichorst, Dimitri V. Meier, Craig W. Herbold, Andreas Richter, Chris Greening, Dagmar Woebken

**Affiliations:** 1 Division of Microbial Ecology, Department of Microbiology and Ecosystem Science, Centre for Microbiology and Environmental Systems Science, University of Vienna, Vienna, Austria; 2 Center for Microbial Communities, Department of Chemistry and Bioscience, Aalborg University, Aalborg, Denmark; 3 Division of Terrestrial Ecosystem Research, Department of Microbiology and Ecosystem Science, Centre for Microbiology and Environmental Systems Science, University of Vienna, Vienna, Austria; 4 Department of Microbiology, Biomedicine Discovery Institute, Monash University, Clayton, VIC 3800 Australia; 5 School of Biological Sciences, Monash University, Clayton, VIC 3800 Australia

**Keywords:** Soil microbiology, Biodiversity, Bacterial physiology

## Abstract

Significant rates of atmospheric dihydrogen (H_2_) consumption have been observed in temperate soils due to the activity of high-affinity enzymes, such as the group 1h [NiFe]-hydrogenase. We designed broadly inclusive primers targeting the large subunit gene (*hhyL*) of group 1h [NiFe]-hydrogenases for long-read sequencing to explore its taxonomic distribution across soils. This approach revealed a diverse collection of microorganisms harboring *hhyL*, including previously unknown groups and taxonomically not assignable sequences. Acidobacterial group 1h [NiFe]-hydrogenase genes were abundant and expressed in temperate soils. To support the participation of acidobacteria in H_2_ consumption, we studied two representative mesophilic soil acidobacteria, which expressed group 1h [NiFe]-hydrogenases and consumed atmospheric H_2_ during carbon starvation. This is the first time mesophilic acidobacteria, which are abundant in ubiquitous temperate soils, have been shown to oxidize H_2_ down to below atmospheric concentrations. As this physiology allows bacteria to survive periods of carbon starvation, it could explain the success of soil acidobacteria. With our long-read sequencing approach of group 1h [NiFe]-hydrogenase genes, we show that the ability to oxidize atmospheric levels of H_2_ is more widely distributed among soil bacteria than previously recognized and could represent a common mechanism enabling bacteria to persist during periods of carbon deprivation.

## Introduction

Soil bacteria consume molecular hydrogen (H_2_) from the Earth’s atmosphere and serve as the main sink in the global biogeochemical H_2_ cycle as demonstrated in field and laboratory-based investigations [1–6]. Hydrogen oxidation in soil follows biphasic kinetics with both high-affinity (*K*_m_ < 100 nM) and low-affinity (*K*_m_ > 1000 nM) enzyme activities [7, 8]. The ability to oxidize H_2_ stems from the presence of specialized metalloenzymes, called hydrogenases, that catalyze the conversion of H_2_ to protons and electrons [9]. Bacteria with low-affinity have been known for decades [10] and are believed to grow on high concentrations of H_2_ produced in microniches, such as N_2_-fixing root nodules [11]. In contrast, bacteria that consume atmospheric H_2_ (~0.53 ppmv) [5] remained elusive until recently [12].

While soil microorganisms harbor a range of hydrogenases that catalyze H_2_ oxidation under oxic conditions [9, 10, 13–16], it is thought that the group 1h [NiFe]-hydrogenases are primarily responsible for (sub-) atmospheric H_2_ oxidation [13, 14, 17], as seen with several previously isolated actinobacteria [9, 12] that consume atmospheric H_2_ to conserve energy during persistence [17–19]. *Actinobacteria* are abundant in soils based on culture-independent studies [9, 14, 20] and were thought to be primarily responsible for atmospheric H_2_ oxidation [14, 16].

Recent genomic and metagenomic investigations, along with pure culture work, have identified additional bacteria harboring group 1h [NiFe]-hydrogenases within the phyla *Acidobacteria*, *Proteobacteria*, *Planctomycetes*, *Chloroflexi* and *Verrucomicrobia* [14, 21–23]. Notably, *Acidobacteria* are one of the most abundant soil phyla with relative abundances in 16S rRNA libraries ranging from ca. 20 to 40% in temperate soils such as forests, grasslands and pasture soils [24]. They constitute a large and phylogenetically distinct phylum [25, 26] that harbors, diverse physiologies [27]. Two thermophilic acidobacterial strains were previously shown to consume atmospheric levels of H_2_, due to the presence of high-affinity [NiFe] hydrogenases [21, 28]. A recent large-scale comparative genome analysis of acidobacteria also identified the genes encoding the large and small subunits of the group 1h [NiFe]-hydrogenase (*hhyL* and *hhyS*, respectively) in genomes of various mesophilic soil acidobacteria, along with the necessary maturation and accessory genes [29]. Yet, it remained unknown whether mesophilic acidobacteria can scavenge H_2_, which are highly abundant in temperate soils where atmospheric H_2_ consumption was previously reported [30–32].

As more and more taxonomic groups have been identified to harbor group 1h [NiFe]-hydrogenases, it was our goal to design broadly inclusive primers for long-read sequencing that allow the investigation of diverse group 1h [NiFe]-hydrogenase communities. Sequencing of almost the complete large subunit gene further enables improved phylogenetic placement and identification of the amplified genes from soil samples. Using this new primer pair, we demonstrate that group 1h [NiFe] hydrogenases are widespread across many phyla, including previously unidentified groups. In addition, we illustrate that mesophilic acido-bacteria are prevalent and active members of the group 1h [NiFe] hydrogenase-harboring community in H_2_-consuming temperate soils and are capable of atmospheric H_2_ consumption. This work therefore reveals new mediators in the biogeochemically and ecologically important process of atmospheric H_2_ oxidation, and supports growing evidence that trace gases might be a universal energy source for bacterial persistence.

## Materials and methods

### Screening publicly available genomes and MAGs

Publicly available genomes and metagenome-assembled genomes (MAGs) (*n* = 175509, November 2018) were screened for the presence of hydrogenase large subunit genes using pfam model PF00374.19, as well as models constructed to be more sensitive to [NiFe] lineages 1–4 from HydDB [33]. For lineage-sensitive models, [NiFe] hydrogenases contained in HydDB were separated into the four major lineages [1–4] and models were constructed *de novo*. Amino acid sequences for each lineage were extracted from HydDB, clustered into centroids using usearch [34] (-sortbylength and –clustersmallmem –id 0.85) and aligned using MAFFT [35]. The resulting alignments were trimmed using trimAl with setting:automated1 [36] and models were constructed using hmmbuild from hmmer3 [37]. [NiFe] hydrogenases were identified in genomes and MAGs using hmmsearch (e-value<0.001). For all hidden Markov model (hmm) hits, the putative genes were back-screened against the Pfam-A database to verify that pfam model PF00374.19 was the best matching Pfam. All putative group 1 [NiFe]-hydrogenase large subunit genes (*hhyL*) were extracted and further screened using the HydDB online classifier [33]. CheckM was used to estimate completeness, contamination and heterogeneity of the genomes based on lineage-specific markers [38]. The taxonomy of MAGs containing the *hhyL* gene with a completeness of >50% was determined using the Genome Taxonomy Database (https://gtdb.ecogenomic.org). Duplicate copies of *hhyL* in a genome were removed and scored as one. The amino acid sequences derived from these *hhyL* genes were used to explore the phylogeny and confirm the taxonomic assignment of the full-length sequences retrieved in this study. Sequences were aligned with MUSCLE [39] and phylogenetic trees were generated using FastTree JTT + CAT model, along with estimating FastTree confidence [40].

### Soil sample collection and nucleic acid extraction

Soil samples were collected from (a) a mature beech forest (*Fagus sylvatica* L.), ca. 40 km southwest of Vienna, Austria (more details can be found in [41]) collected in summers of 2012, 2013, 2014; (b) a managed grassland from the agricultural research station (AREC) in Raumberg-Gumpenstein, Austria (49°29’37”N, 14°06’10”E; more details can be found in [42]) collected in the summer of 2018; (c) the rhizosphere of *Arrhenatherum elatius* (tall oat-grass) grown at this aforementioned managed grassland, collected in the summer of 2018; and (d) biological soil crusts (of ~5 mm thickness) from the central Negev Desert, Israel (30°47’N, 34°46’E; more details on the site can be found in [43]) collected in the summer of 2017. DNA and RNA were extracted from ca. 0.4–0.5 g of soil using a modified bead-beating protocol in the presence of a CTAB buffer and phenol as previously described [44]. Samples were purified using OneStep™ PCR Inhibitor Removal Kit (Zymo, Irvine, CA, USA) and quantified using the Qubit dsDNA BR Assay Kit (Thermo Fisher Scientific, Waltham, MA, USA). For the generation of cDNA, extracts were purified with the Turbo DNA-free kit, quantified with the Qubit HS RNA Assay and reverse-transcribed using SuperScript IV Reverse Transcriptase, all according to the manufacturers’ protocol. All reagents and kits were purchased at Thermo Fisher Scientific, Waltham, MA, USA.

### Sequencing of the group 1h [NiFe]-hydrogenase

Broadly inclusive primers for the large subunit gene (*hhyL*) of the group 1h [NiFe]-hydrogenase were designed using the program CODEHOP [45] using high-quality, full-length sequences downloaded from the HydDB [33] along with acidobacterial sequences (*Acidobacteriaceae* bacterium KBS 83 and *Acidobacteriaceae* bacterium KBS 96, *Granulicella mallensis* strain MP5ACTX8, *Edaphobacter aggregans*, “*Ca*. Solibacter usitatus” Ellin6076) (n = 105 sequences). Primer sequences are listed in Table S1. Reactions were performed in volumes of 25 µl containing the following components: 10 ng to 50 ng of DNA template, 2.5 µl of 10 × DreamTaq Green Buffer, 0.2 mM of each nucleotide dNTP mixture, 0.2 µg µl^−1^ of BSA, 0.5 µM of each primer, and 1.25 U of DreamTaq Green DNA Polymerase (all from Thermo Fisher Scientific, Waltham, MA, USA). Primers were optimized for annealing temperature and cycle number using soil DNA and genomic DNA of *Acidobacteriaceae* bacterium KBS 83. The final PCR program for amplification was: 95 °C for 3 min followed by 38 cycles of 95 °C for 30 s, 65 °C for 30 s and 72 °C for 1.5 min, and a single step of final elongation at 72 °C for 10 min.

Primers were adapted with barcodes at the 5’ end for each primer with 16-nt symmetric barcodes to allow multiplexing of samples in a single run [46]. Positive PCR products were purified using the CleanNA NGS magnetic bead-based clean-up system (CleanNA, Waddinxveen, Netherlands) and sequenced on a PacBio Sequel System (Pacific Biosciences, PacBio, Menlo Park, CA, USA) in circular consensus mode at the Vienna BioCenter Core Facilities (https://www.viennabiocenter.org/facilities/). Consensus sequences were generated for each read with more than five passes of circular sequencing and the reads were demultiplexed using pbbioconda package (https://github.com/PacificBiosciences/pbbioconda). Additional filtering, where average quality within a sliding window of 10 bp should be above 70 and no base call should have quality below 10, was performed with Mothur v. 1.34 [47]. Quality-trimmed sequences longer than 1000 bp were clustered at 95% identity using VSEARCH [48]. The 95% OTU cut-off was used based on the comparison between the sequence similarity scores of group 1h [NiFe]-hydrogenase large subunit genes and the 16S rRNA genes [9]; very few sequences had a similarity above 90%, as such we felt that 95% was a conservative cut-off for this functional gene. This cut-off is also consistent with a recent publication where organisms of the same species had an average nucleotide identity of ≥95% [49]. OTU centroids were taxonomically classified based on diamond blastx [50] search against NCBI-Nr database. Specificity of the amplified sequences was assessed with hmm models of [NiFe]-hydrogenase groups generated with data from HydDB [33]. Diversity estimates and β-diversity were assessed using the phyloseq Package in R [51]. Phylogenetic trees were constructed with OTU representatives based on deduced amino acid sequences in FastTree [40] along with a reference database [33] to identify acidobacterial clusters and closest relatives.

Sister libraries using previously published group 1h [NiFe]-hydrogenase primers [NiFe]-244F/568R and [NiFe]-1129F/1640R [18, 52] (Table S1) were generated on the above soil samples; details can be found in Supplemental Results and Discussion S1-1. The raw sequence data were deposited into the NCBI Short Read Archive under BioProject accession number PRJNA649096.

### Evaluating group 1h [NiFe]-hydrogenase communities across the amplicon libraries

The near full-length group 1h [NiFe]-hydrogenase sequences stemming from PacBio sequencing were compared to the (i) [NiFe]-244F/568R MiSeq-derived sequences and (ii) [NiFe]-1129F/1640R MiSeq-derived sequences using bidirectional nucleotide blast (Nucleotide-Nucleotide BLAST v2.8.1+). Briefly, the top bit scores were generated using a word size of 7, maximum target sequences of 1 and normalized to the respective MiSeq self-blast ([NiFe]-244F/568R or [NiFe]-1129F/1640R). Data were plotted on a frequency histogram to assess the overlap of the communities.

The classification of the OTU representatives was performed using the Evolutionary Placement Algorithm (EPA) implementation in RAxML [53] using the GTR model of amino acid substitution. Briefly, a hmm model was generated based on a MAFFT [35] alignment (L-INS-I mode) of HydDB [33] sequences using hmmer v3.1b2 (www.hmmer.org) [54]; this model was used to align sequences for the base tree construction and to place sequences using EPA. A base tree of 1,716 amino acid reference sequences (avg. 585 amino acids in length) of the group 1h [NiFe]-hydrogenase was generated in FastTree based on the JTT + CAT model [40] upon aligning the sequences using the hmm model (hmmer v3.1.b2). The OTU representatives from the three amplicon libraries ([NiFe]-244F/568R MiSeq; [NiFe]-1129F/1640R MiSeq; [NiFe]-full-length PacBio) were translated and manually curated upon alignment using the hmm model (hmmer v3.1.b2). Poorly aligned sequences were removed from the final dataset leaving 325 (ca. 100 amino acid in length), 61 (ca. 130 amino acid in length) and 1923 (ca. 486 amino acid in length) sequences from [NiFe]-244F/568R MiSeq, [NiFe]-1129F/1640R MiSeq, and [NiFe]-full-length PacBio amplicon libraries, respectively. Maximum likelihood trees were reconstructed in RAxML.

The average genetic distance was determined for the hmm aligned OTU representatives from [NiFe]-244F/568R MiSeq, [NiFe]-1129F/1640R MiSeq and [NiFe]-full-length PacBio amplicon libraries using MEGA7 [55] by computing the pairwise distances. Briefly, the nearly full-length sequences were trimmed to each respective region of the MiSeq primer pairs and pairwise distances were compared using the Poisson model, with a uniform rate among sites. Gaps and missing data were treated as pairwise deletions.

### Bacterial strains and growth conditions

Acidobacterial strains (*Acidobacteriaceae* bacterium KBS 83 [56] and *E. aggregans* [57]) were grown using a defined vitamins and salts medium, VSB-6 [56, 58], with 5 mM glucose as the carbon source. The use of a defined medium allows one to vary total carbon (here suppled as glucose) to determine when the final yield (as measured by optical density at 600 nm) reduced to the proportional decrease in glucose concentration. To determine carbon-limiting conditions, strains were grown in the aforementioned defined medium in differing glucose concentrations (5 mM and 10 mM glucose). Carbon-limiting conditions were defined when the cellular density was proportional to the amount of carbon provided. They were incubated on an orbital shaker (ca. 130 RPM) under aerobic conditions at 24 °C. Growth was monitored by measuring the optical density at 600 nm.

### Primer design and RT-qPCR

Total RNA was extracted from cultures using a modified standard bead-beating protocol [44] with one-round of bead beating and acidified phenol/choloroform/isoamyl alcohol (pH 4.5). Extracts were purified with the Turbo DNA-free kit according to the manufacturer’s protocol. RNAs were normalized to 3 ng µL^−1^ and ca. 30 ng were used for cDNA synthesis using SuperScript IV Reverse Transcriptase, following the manufacturer’s protocol.

Quantitative PCR (qPCR) was performed on a C1000 Touch thermocycler equipped with a CFX96 Real Time System in combination with iQ SYBR-Green qPCR Assay (Biorad, Hercules, CA, USA). Primers were designed for the large (*hhyL*) and small (*hhyS*) subunits of the group 1h [NiFe]-hydrogenase of *E. aggregans* and *Acidobacteriaceae* bacterium KBS 83 targeting the same region of the respective gene, along with the homolog of the *hhyS* as was done previously [21]. All primers are listed in Table S1. Standard curves were constructed with 10-fold serial dilutions of genomic DNA of each respective strain, typically ranging between 10^6^ to 1 copy. The qPCR assay was performed in 20 µl volume containing the following components: 10 µl of SYBR Green Supermix (Biorad, Hercules, CA, USA), 0.4 ng µl^−1^ BSA (Thermo Fisher Scientific, Waltham, MA, USA), 0.2 µM (E. *aggregans*) or 1 µM (*Acidobacteriaceae* bacterium KBS 83) of each primer and 1–5 µl of cDNA template. The program used was: 95 °C for 3 min, followed by 40 cycles of 95 °C for 15 s, 65 °C (*hhyS*, *hhyL* for *Acidobacteriaceae* bacterium KBS 83 were run at 68 °C and 58 °C, respectively) for 30 s for annealing and 72 °C for 1 min for extension. The expression of the large and small subunit genes of the group 1h [NiFe]-hydrogenase was assessed using these newly designed primer pairs upon normalization to the 16S rRNA gene using the above assay but with an annealing temperature of 68 °C. Melting curves were generated between 65 °C and 95 °C. Data were processed and analyzed using the CFX Manager software (Biorad, Hercules, CA, USA) and data were log-transformed to determine fold-increase. Data on the specificity of the qPCR assay can be found in Fig. S1.

### H_2_ consumption and hydrogenase activity measurement assays for acidobacterial cultures

Briefly, cells were harvested in exponential (OD_600nm_ ranging from 0.25–0.38) and stationary (OD_600nm_ ranging from 0.50–0.53) phase by centrifugation at 10000 RPM for 10 min to allow pelleting of these cells that produce extracellular material. Harvested cells were concentrated 10-fold, resuspended in 10 mL carbon-free VSB medium and transferred to 110 mL serum vials sealed with butyl rubber stoppers. Headspace was flushed with synthetic air (Messer Gas, Bad Soden, Germany) and supplemented with ~20 ppmv H_2_ (Linde Gas, Dublin, Ireland). Strains were incubated at 24 °C on an orbital shaker (ca. 130 RPM) for 7 days. Headspace samples were periodically sampled and the H_2_ concentration was determined via a Trace GC Ultra (Thermo Scientific, Austria) with a pulse discharge detector (PDD). This GC has the ability to accurately detect H_2_ down to concentrations of 0.5 ppmv. A gas chromatograph with a pulsed discharge helium ionization detector (model TGA-6791-W-4U-2, Valco Instruments Company Inc. (VICI, Houston, TX, USA)) was used for sub-atmospheric H_2_ concentrations (ca. 0.1 to 0.5 ppmv) as previously described [22]. To ensure an oxygenated headspace during these incubations, oxygen concentrations were monitored and were never lower than 18% (v/v) (Fig. S2). Hydrogen consumption controls were run on uninoculated medium, heat-killed *Acidobacteriaceae* bacterium KBS 83 cells and an acidobacterial strain (*Terriglobus roseus* KBS 63) that does not contain group 1h [NiFe]-hydrogenase genes [29] over a period of 24 h with starting H_2_ concentrations of ~80 ppmv to ensure any potential consumption activity would be sufficiently high for detection (Fig. S3).

The enzyme kinetics (*V*_max_, *K*_m[app]_) of the group 1h [NiFe]-hydrogenase of pure cultures were determined using similar methods as described in [17] using gas chromatography. Briefly, cells were harvested as described above, concentrated and resuspended in carbon-free VSB medium with various concentration of H_2_ (ranging from 0 to 1000 ppmv). Consumption was monitored via gas chromatography over a period of 120 h. H_2_ uptake rates were determined at each respective concentration and normalized to mg of protein using the Pierce BCA Protein Assay Kit (Thermo Fisher Scientific, Waltham, MA, USA). Enzyme kinetics for the Michaelis–Menten kinetics, non-linear least squares method was determined in R [59], while the Hanes-Woolf plot was calculated manually.

### Soil H_2_ consumption assays

Soil samples were collected from aforementioned mature beech forest, managed grassland and desert biological soil crusts to explore H_2_ consumption. Briefly, approximately 1–2 grams of soil (or of natural dry soil crust) was incubated in a sealed 110 mL serum bottle, flushed with synthetic air and supplemented with ~20 ppmv H_2_. H_2_ consumption was monitored in the temperate soils using a Trace GC Ultra (Thermo Scientific, Austria) with a PDD-type detector down to a concentration of 0.5 ppmv; sub-atmospheric H_2_ concentrations (ca. 0.1–0.5 ppmv) were determined on a gas chromatograph with a pulsed discharge helium ionization detector (PDHID) (model TGA-6791-W-4U-2, Valco Instruments Company Inc. (VICI)) as described above. All H_2_ measurements of biological soil crust samples were performed using the VICI gas chromatograph with a PDHID detector.

To determine the *K*_m[app]_ of H_2_ for the beech forest soil, we incubated 1–2 grams of soil in a sealed 110 mL serum bottle. The headspace was flushed with synthetic air and supplemented with H_2_ ranging from 10 to 1000 ppmv, as higher concentrations are needed to estimate *K*_m[app]_ and *V*_max_. The headspace was sampled over 24 h and uptake kinetics were calculated using the Michaelis–Menten kinetics, non-linear least squares method was determined in R [59] and normalized to gram dry soil.

## Results

### Grassland and forest soils harbor active and diverse communities of atmospheric H_2_-oxidizing bacteria

**Fig. 1:**
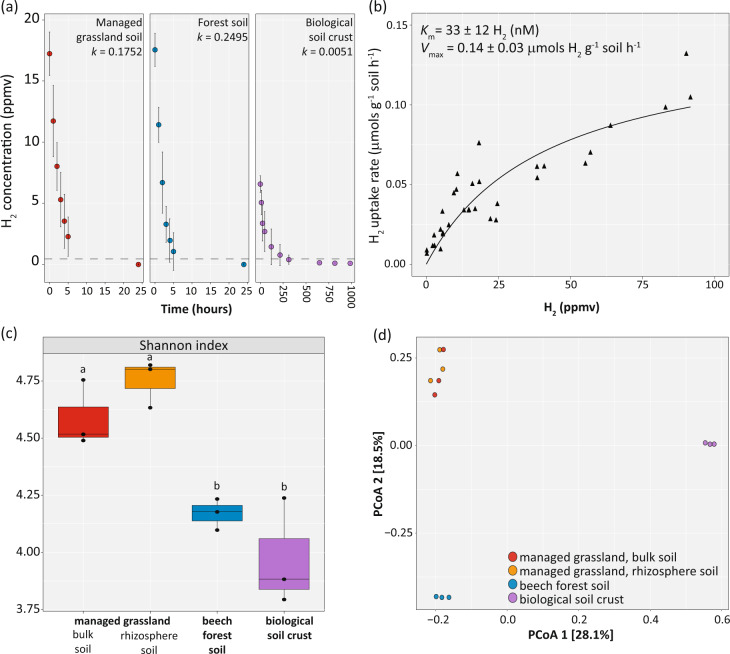
Activity and diversity of bacteria mediating atmospheric H_2_ oxidation in three soil ecosystems. **a** Hydrogen consumption by soils collected from a beech forest, managed grassland and desert biological soil crust. Dotted lines represent atmospheric concentrations of H_2_ (~0.53 ppmv). Data points depict the mean ± standard deviation. **b** Michaelis–Menten kinetics of H_2_ oxidation by the beech forest soil. Best-fit curve was determined using a Michaelis–Menten non-linear regression model. **c** Alpha diversity (Shannon index) and **d** beta diversity (Bray–Curtis dissimilatory) of the group 1h [NiFe]-hydrogenase large subunit (*hhyL*) genes in the soils detected by long-read amplicon sequencing. Analysis of variance with a Tukey’s HSD mean separation was performed across the soil types for the Shannon index; similar letters indicate that no significant difference was observed (*p* value > 0.05). Data were rarefied; unrarefied comparisons can be found in Fig. S4.

We measured bacterial H_2_ consumption in soils from a beech forest, a managed grassland and a desert biological soil crust. The desert soil crust consumed H_2_ very slowly over the course of two weeks to sub-atmospheric levels (Fig. 1a). In contrast, the forest and managed grassland soils rapidly consumed H_2_ to sub-atmospheric levels over a 24-h period (Fig. 1a). Differences between H_2_ uptake amongst the soils are reflected in the contrasting rate constant (*k*) values (Fig. 1a). There was no significant difference in the estimated rate constant values between the temperate soils. The apparent Michaelis constant, *K*_m[app]_, was estimated to be 33 ± 12 nM (Fig. 1b in the beech forest soil); this soil had a significantly (*p* value < 0.05) higher rate constant value when compared to the biological soil crust.

To reveal the microorganisms mediating this H_2_ uptake, we designed degenerate primers to target the group 1h [NiFe]-hydrogenase large subunit genes (*hhyL*) for long-read amplicon sequencing of the different soil communities. These primers were designed to encompass the diversity of *hhyL* sequences across ten different phyla. We amplified and sequenced *hhyL* genes from total community DNA extracted from the managed grassland (bulk and rhizosphere) and forest soils, along with biological soil crusts. Overall, diverse *hhyL*-encoding communities were observed across the soils, with 2403 OTU_95_ clusters (95% cut-off) identified in total. The designed primers proved highly specific for the group 1h [NiFe]-hydrogenase subgroup, as no other genes were amplified (Table S2) and rarefaction curves confirmed the sequencing effort was sufficient to capture the diversity of *hhyL* sequences present (Fig. S4a, Shannon index). Based on the Shannon index, the H_2_-oxidizing community in the managed grassland soils had a significantly higher diversity as compared to the beech forest soil (avg. *p* value < 0.02) and biological soil crusts (avg. *p* value < 0.003), which was observed for rarefied data (Fig. 1c); a similar diversity pattern was observed for the unrarefied data (Fig. S4c). The composition of the *hhyL*-harboring communities was more similar amongst the managed grassland bulk, managed grassland rhizosphere and forest soils than the biological soil crust based on rarefied (Fig. 1d) and unrarefied data (Fig. S4b).

**Fig. 2:**
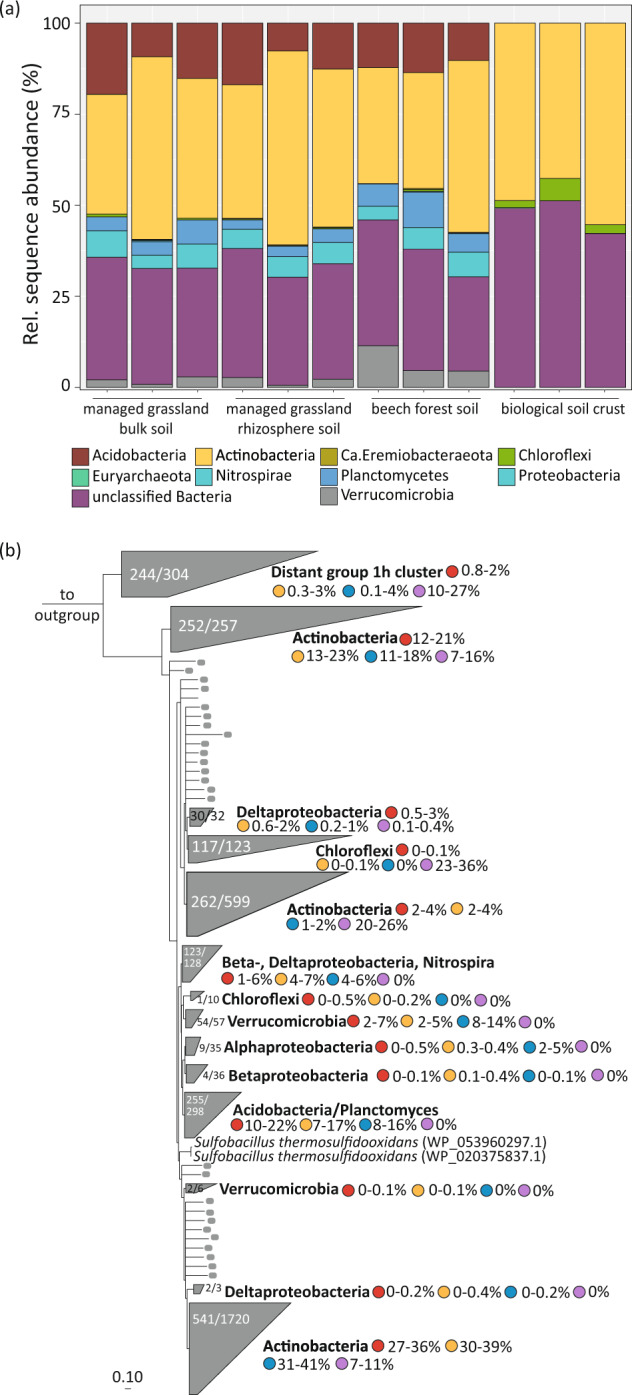
Taxonomic and phylogenetic analysis of the group 1h [NiFe]-hydrogenase large subunit (*hhyL*) genes in three soil ecosystems. **a** Predicted taxonomic distribution of amplified *hhyL* sequences across the investigated soils based on their closest hits to sequences in the NCBI-nr database. Triplicate samples are depicted for each soil. Colors represent different taxonomic groups. **b** RAxML-EPA tree of amino acid sequences of the group 1h [NiFe]-hydrogenase large subunit (*hhyL*) from long-read amplicon sequencing and reference sequences. The phylogenetic placements of OTU representatives stemming from nearly full-length sequences are depicted in gray: collapsed clusters containing OTU representative are shaded gray and non-clustered OTU representatives are colored gray. The number of the placed, nearly full-length *hhyL* sequences and the total number of sequences in each cluster are depicted. The proportions of sequences within each soil are depicted to the right of the clusters managed grassland (red), rhizosphere (orange), beech forest (blue) and biological soil crust (purple). Sequences from group 1g [NiFe]-hydrogenases were used as an outgroup. The scale bar indicates the number of substitutions per site.

**Fig. 3:**
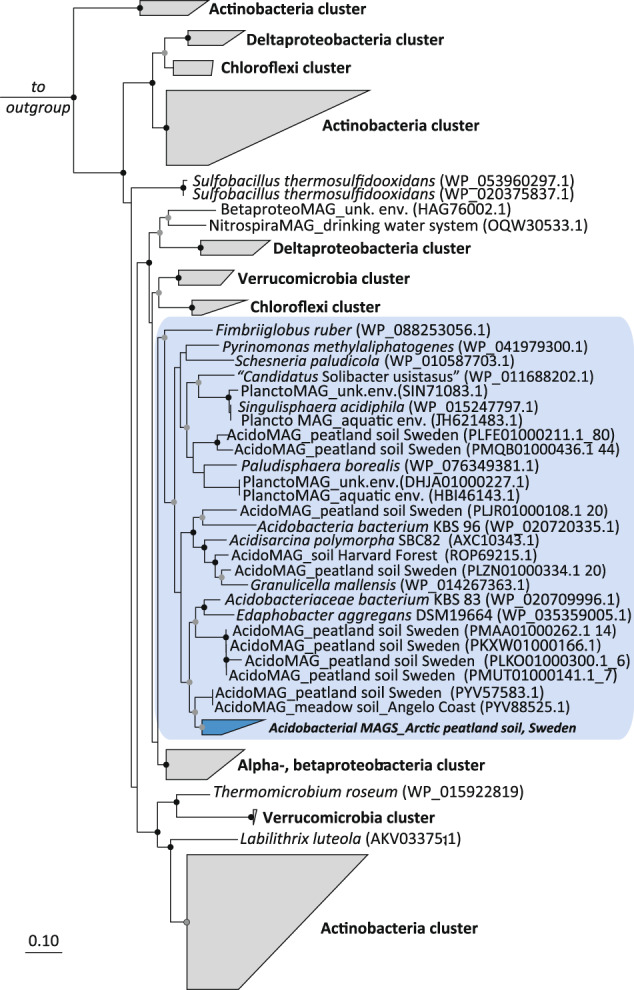
Phylogenetic tree of the group 1h [NiFe]-hydrogenase large subunit (*hhyL*) sequences stemming from reference genomes and metagenome-assembled genomes (MAGs) based on amino acid sequences (*n* = 1650 sequences, ca. 570 amino acid positions). The tree was calculated using FastTree using the JTT + CAT model; FastTree confidence values of >95% (black circles) and >80% (gray circle) are depicted. The scale bar indicates the number of substitutions per site. The acidobacterial cluster is depicted in blue. The environmental source of the sequences (if available) is depicted in the name of each MAG. Additional information on publicly available MAG sequences depicted in the tree can be found in Table S4. Group 1g [NiFe]-hydrogenase sequences were used as an outgroup.

We subsequently performed taxonomic and phylogenetic analysis of the *hhyL* sequences derived from the investigated soils (Fig. 2) alongside those that we retrieved from previously published genomes and metagenome-assembled genomes (Figs. 2 and 3). Bacteria within these temperate soils that encoded group 1h [NiFe]-hydrogenase sequences were affiliated with *Acidobacteria*, *Actinobacteria*, *Chloroflexi*, *Nitrospirae*, *Planctomycetes*, *Proteobacteria* (*Alpha*-, *Beta*- and *Delta*-) and *Verrucomicrobia* (Fig. 2a, b). In contrast, the biological soil crusts harbored group 1h [NiFe]-hydrogenase sequences predominantly affiliated with *Actinobacteria* and *Chloroflexi* (Fig. 2a, b).

There was an additional deep-branching cluster of sequences containing group 1h [NiFe]-hydrogenases of diverse origin, such as members of the phyla *Actinobacteria*, *Chloroflexi*, *Bacteriodetes*, *Acidobacteria*, *Proteobacteria* and *Euryarchaeota* (Fig. 2b, “Distant group 1h cluster”). Although these sequences were all classified as a group 1h [NiFe]-hydrogenase based on the HydDB [33], they appear to be distantly related to the other, main branch of the tree (Fig. 2b). Approximately 244 OTU representatives of the long-read sequences were placed in this cluster; these sequences were found across all soil samples, but were most prevalent in the biological soil crust samples (ca. 10–27%). In addition, there were various OTU representatives that did not group in clusters that contain reference sequences (Fig. 2b, gray dots).

For an evaluation of the newly designed long-read primer pair, we compared its coverage with the coverage of previously published short-read primer pairs ([NiFe]-244F/568R and [NiFe]-1129F/1640R [18, 52]. These short-read primers led to similar patterns of alpha- and beta-diversity (Fig. S5) compared to the long-read primers (Figs. 1c, d and S4). However, the long-read primer pair captured a breadth of diversity exceeding those attained with the short-read primers (Fig. S6). More specifically, the newly designed long-read primer pair captured an additional group of actinobacterial sequences (Fig. S6c, top-most *Actinobacteria* cluster), along with putative members in the *Deltaproteobacteria*, *Nitrospira* and the deep-branching cluster of group 1h [NiFe]-hydrogenases of diverse origin (Fig. S6c, “Distant group 1h cluster”). A further difference amongst the primer pairs was the increased number of sequences without any reference sequences in the long-read amplicon libraries (Fig. S6c, indicated by gray dots) compared to both short-read primer pair sets (Fig. S6a, b). The increased diversity in the long-read primer pair amplicon libraries was further supported by calculating the average genetic distance in the group 1h [NiFe]-hydrogenase community, which was increased compared to the short-read primer pairs (Supplementary Information SI-1), and via bidirectional nucleotide blast analyses and primer match analyses (Supplementary Information SI-1, Fig. S7, Table S3). Taken together, it appears that the newly designed long-read primers pair targets a wider (and putatively novel) phylogenetic diversity of group 1h [NiFe]-hydrogenases in the investigated soils.

### A cluster of acidobacterial high-affinity hydrogenases are abundant and expressed in the grassland and forest soils

The long-read amplicon data further revealed that, after *Actinobacteria*, *Acidobacteria* were the second most represented phylum in the *hhyL* amplicons (Fig. 2). Seven to 22% of amplicon sequences stemming from forest and managed soils were most closely related to *hhyL* sequences from acidobacterial genomes (Fig. 2b). In contrast, acidobacterial hydrogenases were absent from the biological soil crusts (Fig. 2), where over half of the obtained OTUs were affiliated with three actinobacterial-specific *hhyL* clades (Fig. 2b). Analysis of publicly available acidobacterial genomes and MAGs confirmed that ~7% (of 745 genomes) harbored the *hhyL* gene (Fig. S8), including members from subdivisions 1 (genera *Acidipila*, *Edaphobacter* and *Granulicella*), 2 (MAGs only), 3 (genera *Bryobacter*, ‘*Candidatus* Solibacter’) and 4 (genus *Pyrinomonas*). The acidobacterial *hhyL* sequences stemming from pure cultures [56, 57, 60–63], genomes and MAGs originated from soil (Table S4) and formed a distinct cluster (Fig. 3), as also observed with our derived amplicon sequences (Fig. 2b), together with *hhyL* sequences from *Planctomycetes*. Overall the acidobacterial cluster had an average sequence similarity of ca. 83% based on amino acid sequences. Despite the described more limited diversity covered with the short-read primers (Supplementary Information SI-1), amplification of cDNA derived from the soils with these primers revealed that the *hhyL* genes of various phyla, including the *Acidobacteria*, were expressed in the temperate soils (Supplementary Information SI-1, Figs. S9–10).

### Acidobacteria isolated from temperate forest and grassland soils oxidize atmospheric H_2_ during carbon limitation

The above culture-independent inferences suggest that members of the *Acidobacteria* may be important mediators of atmospheric H_2_ oxidation in temperate soils. As such, we investigated the gene expression and activity of the group 1h [NiFe]-hydrogenase present in two mesophilic acidobacterial isolates, the grassland soil bacterium *Acidobacteriaceae* bacterium KBS 83 [56] and forest soil bacterium *Edaphobacter aggregans* [57]. In addition to containing *hhyL* and *hhyS* [29], both organisms also possess an additional copy of *hhyS* ca. 3,600 bp upstream of the structural and maturation genes, as observed in *P. methylaliphatogenes* [21] (Supplementary Information SI-2).

**Fig. 4:**
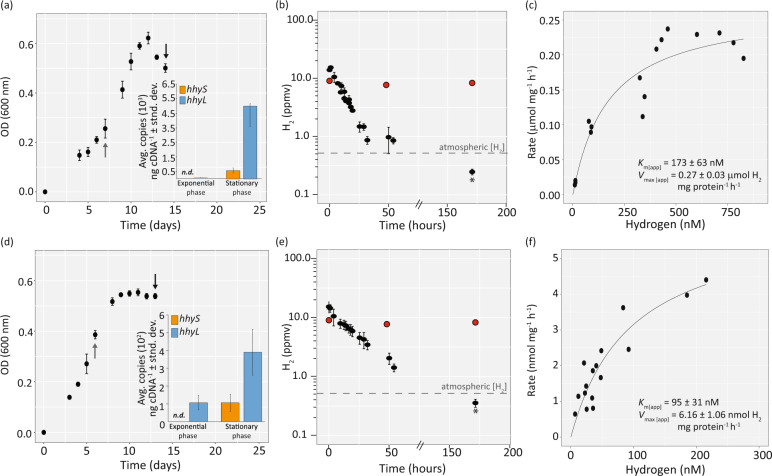
Expression, activity, and kinetics of the enzymes mediating atmospheric H_2_ oxidation in two acidobacterial strains isolated from temperate soils, *Acidobacteriaceae* bacterium KBS 83 and *Edaphobacter aggregans*. **a**, **d** Growth curves of the strains over time (days) (*x*-axis) and expression levels (inset) of the group 1h [NiFe]-hydrogenase structural subunit genes (large, *hhyL*; small, *hhyS*) during exponential and stationary phase. Arrows depict the growth phases in which cells were harvested for gene expression investigations (gray arrow, exponential phase; black arrow, stationary phase). During this experiment, H_2_ consumption was measured on stationary phase cells (black arrows) and in a parallel experiment on exponential phase cells (Fig. S13), as H_2_ consumption assays required the entire biomass of such an experiment. **b**, **e** H_2_ consumption of stationary phase stage cells of each respective strain; *x*-axis depicts the start of measurements for H_2_ consumption after harvesting cells from growth curves of panels **a**, **d**. Dashed lines represent atmospheric H_2_ concentrations (~0.53 ppmv), whereas red points depict the heat-killed controls for each respective strain. The final sub-atmospheric H_2_ measurement for each strain was performed on a gas chromatograph with a pulsed discharge helium ionization detector (model TGA-6791-W-4U-2, Valco Instruments Company Inc.); this measurement is indicated by an asterisk. Over the course of the experiment, we observed a slight decrease in H_2_ for our medium control (8%). Even when this loss is accounted for in the final sub-atmospheric measurement, the concentration is still below atmospheric levels of H_2_, (0.27 ppmv for *Acidobacteriaceae* bacterium KBS 83 and 0.39 ppmv for *E*. *aggregans)*. Additional controls can be found in Fig. S3. **c**, **f** Apparent kinetic parameters of H_2_ oxidation for the strains based on whole-cell assays. Best-fit curves were determined using the Michaelis–Menten non-linear regression model; similar values were observed using Hanes–Woolf plots (Table S5).

Transcription of both the large (*hhyL*) and small (*hhyS*) subunits of the hydrogenase was upregulated during carbon-limitation (Fig. 4a, d). We used defined media to determine the conditions that induced stationary phase due to carbon-limitation in both strains (Fig. S11). Under stationary phase conditions, the transcription of the *hhyL* gene (normalized to the 16S rRNA gene) was upregulated by ~125-fold in *Acidobacteriaceae* bacterium KBS 83 (Fig. 4a) and ~3.5-fold in *E. aggregans* (Fig. 4d) compared to exponential growth. In both organisms, transcription of the *hhyS* gene was not detected during exponential phase (< 2 copies per ng cDNA), but was detected during stationary phase (Fig. 4a, d). The apparent differential expression of the *hhyS* and *hhyL* genes in exponential phase may be attributed to different promoter regions; potential different promoter regions were computationally identified across the structural genes of *Acidobacteriaceae* bacterium KBS 83, which presumably also have different transcription factors (Fig. S12). The expression of the *hhyS* homolog was detected in *Acidobacteriaceae* bacterium KBS 83, but not in *E. aggregans* (Supplementary Information SI-2).

**Fig. 5:**
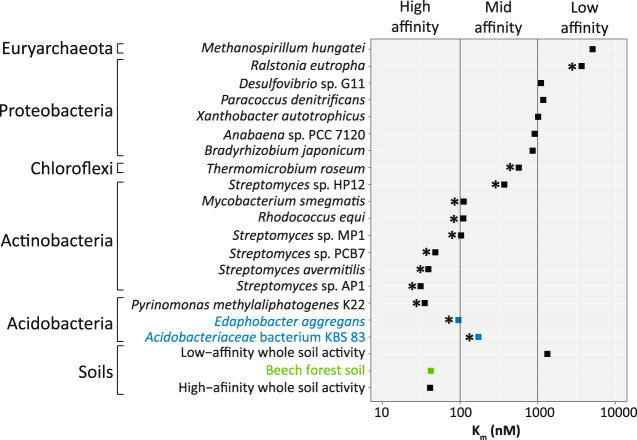
Comparison of apparent substrate affinity (*K*_m[app]_, in nM) for H_2_-oxidizing bacteria and archaea spanning different taxonomic groups. Figure was amended from [17]; data were extracted from Greening et al. [17] and Islam et al. [22]. Asterisks depict microorganisms harboring a group 1h [NiFe]-hydrogenase. Acidobacteria estimates derived in this study are depicted in blue, and data from the investigated beech forest soil are shown in green.

We subsequently tested whether these strains consume atmospheric H_2_ under carbon-limiting conditions. Both strains consumed H_2_ from levels of ~20 ppmv to a concentration of 0.25 ± 0.03 ppmv (*Acidobacteriaceae* bacterium KBS 83) and 0.36 ± 0.06 ppmv (*E. aggregans*) after 172 h (Fig. 4b, e). H_2_ was only consumed by carbon-limited stationary cells; no H_2_ oxidation was observed in cells exponentially growing on the defined medium (Fig. S13) or in *E. aggregans* cultures grown to stationary phase on an undefined medium (presumably due to non-carbon-limiting conditions) (Fig. S14). Likewise, no H_2_ was consumed in the uninoculated medium, heat-killed controls, or in an acidobacterial strain lacking the group 1h [NiFe]-hydrogenase (*Terriglobus roseus*) (Fig. S3). The kinetic parameters of the group 1h [NiFe]-hydrogenases were determined on whole cells of strains *Acidobacteriaceae* bacterium KBS 83 and *E. aggregans*. The apparent half-saturation constant (*K*_m[app]_) measured on cells of *Acidobacteriaceae* bacterium KBS 83 was 173 ± 63 nM H_2_ with a saturating rate (*V*_max[app]_) of 0.27 ± 0.03 µmol H_2_ mg protein^−1^ h^−1^ (Fig. 4c). The *K*_m[app]_ for H_2_ uptake by *E. aggregans* was 95 ± 31 nM with a *V*_max[app]_ of 6.16 ± 1.06 nmol H_2_ mg protein^−1^ h^−1^ (Fig. 4f); similar values were observed using another model (Table S5). These kinetic parameters are consistent with previous estimates for mid- to high-affinity hydrogenases of pure cultures (Fig. 5) and temperate soils, where the *Acidobacteria* are typically found.

## Discussion

Our genomic surveys and the targeted *hhyL* amplicon sequencing of soil samples add to the growing evidence that group 1h [NiFe]-hydrogenases appear to be widespread in numerous taxonomic groups (Fig. 2). The genomic survey identified new groups, namely members of the *Deltaproteobacteria* and *Nitrospira*, that harbor group 1h [NiFe]-hydrogenase genes, in addition to *Acidobacteria*, *Actinobacteria*, *Bacteriodetes*, *Chloroflexi*, *Planctomycetes*, *Proteobacteria* (*Alpha*- and *Beta*-) and *Verrucomicrobia* (Figs. 2 and S8) in accordance with previous surveys [9, 10, 14, 16]. These phyla are commonly found in soils with varying relative abundances [64]; however, it should be noted that with the exception of the *Acidobacteria* and *Actinobacteria*, group 1h [NiFe]-hydrogenases are present in less than 3% of genomes from each of these phyla (Fig. S8). Furthermore, all of the mentioned phyla are amplifiable with our newly designed primers (Fig. 2), thus allowing future investigations to explore the distribution of these groups across environments using long-read amplicon sequencing.

Our newly designed group 1h [NiFe]-hydrogenases primers not only capture the diversity of previously established group 1h [NiFe]-hydrogenases primers [18, 52], but also additional sequence diversity across edaphically different soils (Fig. S6). In comparison to these other primers, phylogenetic analysis revealed the presence of additional clusters as well as OTUs without reference sequences (Fig. S6, in gray), the latter suggesting the presence of putative novel hydrogenases. We suggest that the use of long-read *hhyL* sequences could allow for improved phylogenetic placement and identification of the amplified sequences from environmental samples, along with the use of the Evolutionary Placement Algorithm implemented in RAxML using a base tree built with not only publicly available *hhyL* sequences (mostly stemming from isolates) but also with *hhyL* genes identified in genomes and MAGs of uncultured organisms (Fig. S8). As such, we have added an additional primer pair to the toolbox of exploring the group 1h [NiFe]-hydrogenases in environmental samples. Although it is unclear if it captured the complete diversity of atmospheric H_2_-oxidizers harboring group 1h [NiFe]-hydrogenases in the investigated soils, it captured some putative novel sequences and previously undetected groups (Fig. 2). These findings will aid future work to attribute which community members and enzyme lineages are responsible for the biogeochemically important process of atmospheric H_2_ oxidation.

Biological soil crusts exhibited the lowest diversity estimates (Fig. 1c), being dominated by *hhyL* sequences assigned to *Actinobacteria*, *Chloroflexi* and the “Distant group 1h cluster” (Fig. 2b), and the slowest H_2_ consumption (Fig. 1a, *k* = 0.0051). Temperate soil libraries had higher diversity estimates, especially in the managed grassland soil (Fig. 1c), and contained sequences related to the *Actinobacteria*, *Acidobacteria*, *Planctomycetes*, *Proteobacteria* and *Verrucomicrobia* (Fig. 2). These soils exhibited faster H_2_ consumption (Fig. 1a**;** managed grassland *k* = 0.1752, forest soil *k* = 0.2495). Follow-up investigations will be necessary to reveal the contributions of these different groups to H_2_ oxidation (also including hydrogenases other than the group 1h-type), but preliminary sequencing of cDNA shows a diverse community actively transcribing *hhyL* genes in the temperate soils (Fig. S10).

Many of the *hhyL* sequences detected by using the new primer pair clustered with the two acidobacterial strains tested here for H_2_ consumption (*Acidobacteriaceae* bacterium KBS 83 and *E. aggregans*), in addition to other mesophilic acidobacteria such as *Acidobacteriaceae* bacterium KBS 96, *G. mallensis*, and “*Ca*. Solibacter usitatus” Ellin6076”. All of these strains are described as aerobic heterotrophs [56, 57, 61, 65], which is consistent with the group 1h [NiFe]-hydrogenase being both oxygen-tolerant [66] and linked to the aerobic respiratory chain [10]. The acidobacterial group 1h [NiFe]-hydrogenase sequences from genomes and MAGs formed a distinct clade (Figs. 2 & 3) together with those from the *Planctomycetes*. Some of these *Planctomycetes*-affiliated sequences were generated from MAGs and, as such, this clustering could be a result of poor assemblies, incompleteness or high contamination of MAGs. Yet it is unlikely as this cluster also contains sequences stemming from pure cultures (such as *Singulisphaera acidiphila*) (Fig. 3).

We demonstrate that mesophilic acidobacteria are capable of scavenging H_2_ in pure culture (Fig. 4). Although previous work has shown that two thermophilic acidobacterial strains scavenge atmospheric H_2_ either due to a group 1h or 1f [NiFe]-hydrogenase [21, 28], only very few sequences with an identity of ≥97% could be detected in the NCBI database and of those identified, were primarily from thermophilic environments (Fig. S15). Therefore, investigating the H_2_-oxidation capability in representative mesophilic strains inhabiting temperate soils was essential. With the data presented in this study on mesophilic acidobacteria, this is first report of a mesophilic bacterium outside the *Actinobacteria* [9, 20, 67] being capable of atmospheric H_2_ oxidation via a group 1h [NiFe]-hydrogenase. We were further able to detect expressed acidobacterial *hhyL* in temperate soils (Fig. S10), illustrating that acidobacteria are active in the soils that consume H_2_ (Fig. 1a). This is ecologically significant given mesophilic acidobacteria are abundant across temperate soils [68], which together comprise ~20–30% of terrestrial environments [69]. Further investigations are warranted to determine the acidobacterial contribution to H_2_ consumption in temperate soils, for instance in comparison to the well-studied actinobacteria. The two investigated strains, *Acidobacteriaceae* bacterium KBS 83 and *E. aggregans*, exhibit mid to high-affinity H_2_ uptake kinetics comparable to those measured for other bacteria harboring group 1h [NiFe]-hydrogenases (Fig. 5, asterisks). The whole soil communities within the beech forest soil also exhibited high-affinity H_2_ uptake kinetics (Fig. 1b), suggesting that H_2_ is predominantly being oxidized by bacteria expressing high-affinity enzymes (i.e., group 1h [NiFe]-hydrogenases). This is congruent with previous surveys showing these enzymes are the most prevalent hydrogenases in grassland and forest soils [14].

The *K*_m[app]_ of the investigated mesophilic soil acidobacteria (95 and 172 nM) was higher compared to the thermophilic acidobacterium *P. methylaliphatogenes* (35 nM), but in line with the higher-end of model high-affinity H_2_-oxidizing bacteria such as representatives in the *Actinobacteria* (Fig. 5). Yet the *K*_m[app]_ of the investigated strains was lower than the *Chloroflexi* strain *Thermomicrobium roseum* that consumes H_2_ from both geological and atmospheric sources [22] (Fig. 5). This further demonstrates that group 1h [NiFe]-hydrogenases spanning both the high- and mid-affinity *K*_m[app]_ values are capable of using atmospheric concentrations of H_2_. The *V*_max_ rate for *Acidobacteriaceae* bacterium KBS 83 were ca. 5-fold higher than that observed for *P. methylaliphatogenes* [21], but 2 to 6-fold lower than those observed for *T. roseum* [22] and *Mycobacterium smegmatis* [17]. In contrast, the *V*_max_ rate of *E. aggregans* was orders of magnitude lower than the aforementioned strains. This observed variation in *V*_max_ within the strains used in this study and those previously published [17, 21] suggest there is high variability among group 1h [NiFe]-hydrogenases. Furthermore, the relative contributions of group 1h [NiFe]-hydrogenases to atmospheric H_2_ uptake in-situ remain unclear, as does to what extent duration of carbon starvation or other growth-limiting conditions influences group 1h [NiFe]-hydrogenases expression and potential activity in soil environments.

Our data suggest that mesophilic acidobacteria use atmospheric H_2_ to adapt to carbon starvation, which is in accordance with other soil strains [16, 17]. The structural genes encoding the hydrogenases were highly upregulated by both species in stationary-phase, carbon-starved cultures compared to exponentially growing, carbon-replete cultures (Fig. 4a, d). Likewise, activity was only observed in carbon-starved cultures (Figs. 4b, e and S14). Exponential bacterial growth is a state rarely found in nature; rather in many ecosystems (such as soil), bacteria enter a non-replicative persistent state [70] for extended periods of time [71]. It was estimated that up to 80% of the soil microorganisms at a given time [72] will be in such a state, often referred to as dormancy. Hydrogen has previously been proposed as a possible universal energy source for survival [73]. It was then proposed more specifically that the use of group 1h [NiFe]-hydrogenases facilitate the ability to scavenge atmospheric H_2_ to sustain aerobic respiration during periods of starvation in actinobacteria [17] and thermophilic acidobacteria [21, 28]. In this study, we further extend this working hypothesis to encompass mesophilic acidobacteria. It is likely that the oxidation of atmospheric H_2_, as a ubiquitous, diffusible, high-energy substrate, enables mesophilic acidobacteria to meet maintenance energy needs during persistence [10, 18]. H_2_-scavenging acidobacteria could have a selective advantage in periods of carbon depletion and therefore an increased likelihood to persist in the soil. Although group 1h [NiFe]-hydrogenase genes could only be detected in ca. 7% of 745 investigated acidobacterial genomes (Fig. S8), this may reflect the lack of adequate representations of *Acidobacteria* in our public databases relative to the breadth of diversity found in nature. It is also plausible to assume that there are other physiologies that allow persisting periods of carbon starvation, such as atmospheric carbon monoxide oxidation as suggested recently for actinobacteria [74] and chloroflexi [22].

Overall, the finding that mesophilic acidobacteria and likely other diverse microorganisms in soil can oxidize atmospheric H_2_ has important implications for both atmospheric chemistry and microbial ecology. These bacteria potentially contribute to the major sink of the global H_2_ cycle. Moreover, atmospheric H_2_ scavenging is hypothesized to contribute to bacterial persistence, with theoretical estimates predicting that atmospheric H_2_ provides the necessary maintenance energy for 10^7^ to 10^8^ bacteria per gram of soil [10, 18]. This physiology could, therefore, aid in maintaining populations and genotypes, ultimately sustaining soil microbial biodiversity.

## Supplementary information

Giguere_Eichorst_SOM
