# Supplementary material for: Acidobacteria are active and abundant members of diverse atmospheric H2-oxidizing communities detected in temperate soils
Source: ISME J. 2020 Oct 6;15(2):363–76. doi: 10.1038/s41396-020-00750-8 (PMC8027828; doi:10.1038/s41396-020-00750-8)
Supplement: Supplementary file 1 — Giguere_Eichorst_SOM [file 41396_2020_750_MOESM1_ESM.pdf]

Supplementary Information for

Acidobacteria are active and abundant members of diverse atmospheric H_2-oxidizing communities detected in temperate soils

Andrew T. Giguere, Stephanie A. Eichorst*, Dimitri Meier, Craig W. Herbold, Andreas Richter, Chris Greening, Dagmar Woebken

*Corresponding author: Stephanie A. Eichorst
E-mail address for correspondence: stephanie.eichorst@univie.ac.at

This PDF file includes:
Supplementary text
Figures S1-S15
Tables S1-S5

Supplementary Information

SI-1. Benchmarking nearly full-length group 1h [NiFe]-hydrogenase libraries to parallel shorter read libraries.

Materials and Methods

Sister libraries using previously published group 1h [NiFe]-hydrogenase primers ([NiFe]-244F/568R and [NiFe]-1129F/1640R (1, 2) **Table S1**) were generated on the investigated soil samples. Briefly, DNA and RNA were extracted from ca. 0.4-0.5 g of soil using a modified bead-beating protocol in the presence of a CTAB buffer and phenol as previously described (3). Samples were purified using OneStep™ PCR Inhibitor Removal Kit (Zymo, Irvine, CA, USA) and quantified using the Qubit dsDNA BR Assay Kit (Thermo Fisher Scientific, Waltham, MA, USA). For the generation of cDNA, extracts were purified with the Turbo DNA-free kit, quantified with the Qubit HS RNA Assay and reverse-transcribed using SuperScript IV Reverse Transcriptase, all according to the manufacturer's protocol. All reagents and kits were purchased at Thermo Fisher Scientific, Waltham, MA, USA.

The primers were adapted to include a universal 16 bp head sequence at their 5' end for subsequent barcoding, as described previously (4). The first-step PCR were performed in 25 µl volume containing the following components: 10-50 ng of DNA template, 2.5 µl of 10x DreamTaq Green Buffer, 1 mM $MgCl_2$ (only for [NiFe]-244F, [NiFe]-568R), 0.2 µM of each nucleotide dNTP mixture, 0.2 µg μl^{-1} of BSA, 0.5 µM of each primer, and 1.25 U of DreamTaq Green DNA Polymerase (all from Thermo Fisher Scientific, Waltham, MA, USA). Primers were optimized for annealing temperature and cycle number using soil DNA. The final first-step PCR program for amplification was: 94 °C for 3 min followed by 45 ([NiFe]-244F, [NiFe]-568R) or 30 ([NiFe]-1129F, [NiFe]-1640R) cycles of 94 °C for 35 s, 63 °C ([NiFe]-

244F, [NiFe]-568R) or 68 °C ([NiFe]-1129F, [NiFe]-1640R) for 30 s and 72 °C for 45 s, and a single step of final elongation at 72 °C for 10 min.

Samples were purified using the ZR-96 DNA Clean-up Kit™ (Zymo, Irvine, CA, USA) and 3 µl from the purified sample was used for a second PCR reaction, which was 50 µl in volume and contained the following mixture: 5 µl of 10 × DreamTaq Green Buffer, 0.2 mM of each nucleotide dNTP mixture, 0.2 µg $µl^{-1}$ of BSA, 1.25 U of DreamTaq Green DNA Polymerase (all from Thermo Fisher Scientific, Waltham, MA, USA) and 0.8 µM of a barcode primer, which also contained a universal 16 bp head sequence at the 5' end as described in (4). The final PCR program for amplification was: 94 °C for 4 min followed by 8 cycles of 94 °C for 30 s, 52 °C for 30 s and 72 °C for 45 s, and a single step of final elongation at 72 °C for 10 min. Second-step PCR products were purified using the ZR-96 DNA Clean-up Kit™ (Zymo, Irvine, CA, USA) and quantified using the Quant-iT™ PicoGreen® dsDNA Assay Kit (Thermo Fisher Scientific, Waltham, MA, USA) on a Tecan Safire plate reader (Tecan, Männedorf, Switzerland).

Samples were pooled to equimolar amounts of 20×10^9 copies per sample, followed by library preparation and sequencing provided by Microsynth AG (Balgach, Switzerland). The library was prepared by adapter ligation and PCR using the TruSeq Nano DNA Library Prep Kit (Illumina, Cat FC-121-4001) according to the TruSeq nano protocol (Illumina, FC-121-4003), excluding the fragmentation step. Sequencing was performed on a MiSeq platform (Illumina, San Diego, CA, USA), in the 2 × 300 cycle configuration using the MiSeq Reagent kit v3 (Illumina, San Diego, CA, USA). Paired MiSeq reads were split into sample datasets as described previously (3). The 95% OTU cut-off was used based on the comparison between the sequence similarity scores of group 1h [NiFe]-hydrogenase large subunit gene and the 16S rRNA gene (5); very few sequences had a similarity above 90%, as such we felt that 95% was a conservative cut-off for this functional gene. This cut-off is also consistent with a recent publication where organisms of the same species had an average nucleotide identity of ≥95% (6). As such, we took this average identity value as a threshold to generate OTUs for each respective primer pair ([NiFe]-244F/568R and [NiFe]-1129F/1640R).

Results and Discussion

Comparison of the group 1h [NiFe]-hydrogenase DNA-based libraries

We evaluated the group 1h [NiFe]-hydrogenase community stemming from the newly designed long-read primers and the previously published primers ([NiFe]-244F/568R and [NiFe]-1129F/1640R (1, 7)) across beech forest soil, managed grassland soil and biological soil crusts. The [NiFe]-244F/568R primer pair generated 163,855 reads that clustered into 338 OTU_{95} (95% cut-off), whereas the [NiFe]-1129F/1640R generated 39,453 reads that clustered into 66 OTU_{95}. Across both smaller fragment libraries, the soils were fairly well-sampled based on rarefaction curves (**Fig. S5a,b,** Shannon index). The group 1h [NiFe]-hydrogenase communities were distinct across the investigated soils based on

NMDS using the Bray-Curtis dissimilarity index (**Fig. S5c,d**), as observed in the long-read amplicon libraries (**Fig. 1d** (rarefied data), **S4b** (unrarefied data)). The diversity and richness were typically higher in the temperate soils (beech forest and managed grassland soils) relative to the biological soil crusts (**Fig. S5e,f**). These diversity and richness patterns were similar to the long-read amplicon libraries when data were rarefied and unrarefied (**Fig. S4c,d**).

Although similar alpha- and beta-diversity patterns amongst soils were observed across the 3 investigated primer pair sets, we observed clear differences in the phylogenetic affiliation of the OTU representatives using RAxML EPA trees (**Fig. S6** – comparison of the grey shaded clusters and dots). The OTU representatives stemming from the [NiFe]-244F/568R primer pair spanned numerous phylogenetic phyla based on RAxML EPA, such as *Actinobacteria*, *Proteobacteria*, *Verrucomicrobia*, *Acidobacteria*, *Planctomycetes* and *Chloroflexi* (**Fig. S6a**), while OTU representatives stemming from [NiFe]-1129F/1640R amplicon library were identified as *Actinobacteria*, except two OTU representative (**Fig. S6b**). The OTU representatives of the nearly full-length PacBio amplicon libraries also spanned numerous phylogenetic phyla based on RAxML EPA, such as *Actinobacteria*, *Proteobacteria*, *Verrucomicrobia*, *Acidobacteria*, *Planctomycetes*, *Chloroflexi* (**Fig. S6c**), but appeared to capture additional members of the group 1h [NiFe]-hydrogenase community not captured by the [NiFe]-244F/568R primer pair, including putative members of the *Deltaproteobacteria* and *Nitrospira*. This newly designed primer pair also covered an additional group of actinobacterial sequences (**Fig. S6c**, top-most Actinobacteria cluster), as well as an additional deep-branching cluster of sequences containing group 1h [NiFe]-hydrogenases of diverse origin, such as members of the *Actinobacteria*, *Chloroflexi, Bacteriodetes, Acidobacteria, Proteobacteria* and *Euryarchaeota* (**Fig. 6c**, "Distant group 1h cluster"). Although these sequences were all classified as a group 1h [NiFe]-hydrogenase based on the HydDB (8), they appear to be distantly related to the other, main branch of the tree (**Fig. 2b**). Furthermore, we detected numerous sequences that were placed in the tree without reference sequences (**Fig. S6c**, indicated by grey dots) in the long-read amplicon libraries, in comparison to 1 ([NiFe]-1129f/1640R) or 5 ([NiFe]-244f/568R) OTU representatives without reference sequences in the short-read libraries (**Fig. S6a-c** – comparison gray shading). Taken together, it appears that these new primers target a wider (and putative novel) phylogenetic diversity of group 1h [NiFe]-hydrogenases.

This increased diversity in the long-read amplicon libraries was further supported by assessing the average genetic distances. The average pairwise genetic distance among the nearly full-length sequences was ca. 48% (when trimmed to the [NiFe]-244F/568R region) or 50% (when trimmed to the [NiFe]-1129F/1640R region), compared to 18% ([NiFe]-1129F/1640R) or 25% ([NiFe]-244F/568R) from the shorter amplicon sequences. A similar pattern was observed for the genetic distance of the acidobacterial/plancomycetes sequences: nearly full-length sequences showed genetic distance of ca. 23% (when trimmed to the [NiFe]-244F/568R region), compared to sequences stemming from [NiFe]-

244F/568R amplicon libraries (distance of ca. 17%) suggesting more of the acidobacterial community was captured with this new primer pair.

Bidirectional nucleotide blast analyses were performed on the OTU representatives amongst the amplicon libraries to evaluate the similarity of the amplified group 1h [NiFe] hydrogenase sequences amongst the 3 primer pairs. Bidirectional nucleotide blast analysis allows one to ascertain the proportion of sequences that are homologous between the 2 investigated sequence sets. If the two investigated sequence sets are identical, a completely overlapping histogram will be observed. However, if the two investigated sequence sets are not identical, the histograms will appear shifted or skewed illustrating that different sequence representatives are present in each respective sequence set.

When OTU representatives of [NiFe]-244F/568R and [NiFe]-1129F/1640R Illumina-based sequences were mapped to long-read OTU representatives, the distribution of both histograms stemming from sequence sets of [NiFe]-244F/568R and [NiFe]-1129F/1640R were skewed to the right (**Fig. S7**, purple bars). The bit scores (reflection of similarity) were high. Taken together, this illustrates that more of the shorter sequences aligned to the long-read sequences. In contrast, when the long-read OTU representatives were mapped to OTU representatives of the [NiFe]-244F/568R and [NiFe]-1129F/1640R Illumina-based sequences, there was a bimodal distribution, skewed to the right upon (**Fig. S7**, blue bars). The bit scores (reflection of similarity) were lower. Taken together, these data indicate that less of the PacBio sequence representatives aligned to the smaller, Illumina-based representatives.

These data suggest that the long-read primers for the group 1h [NiFe]-hydrogenases not only captured the breadth of diversity one can attain with the primer sets, [NiFe]-244F/568R and [NiFe]-1129F/1640R, but also captured additional diversity. This is further supported when searching for the primer regions of the [NiFe]-244F/568R and [NiFe]-1129F/1640R in the PacBio sequences. The majority of the long-read OTU representatives (ca. 46 to 85%) had more than 4 mismatches for the priming regions of [NiFe]-244F/568R and [NiFe]-1129F/1640R (**Table S3**). As these priming regions could not be found in the majority of long-read sequence representatives without numerous mismatches, it further illustrates that this newly designed primer set targets additional group 1h [NiFe]-hydrogenase diversity in these environmental samples.

<u>cDNA libraries of the group 1h [NiFe]-hydrogenase</u>

The [NiFe]-244F/568R primer pair generated 828,149 reads that clustered into 110 OTU_{95} (95% cut-off) stemming from cDNA-based libraries. The soils were fairly well-sampled based on rarefaction analysis (**Fig. S9a**). Unlike the DNA-based libraries stemming from the [NiFe]-244F/568R primer pair as well as the long-read amplicon libraries, the group 1h [NiFe]-hydrogenase communities did not

separate based on soil type (**Fig. S9b**). This suggests that a diverse collection of microorganisms were expressing their *hhyL*, which was not (directly) influenced by the edaphic properties of the investigated soils. The diversity and richness were typically higher in the managed grassland soils relative to the beech forest soil (**Fig. S9c**). The rhizosphere soil diversity was lower than of the bulk soil from the managed grassland soil (**Fig. S9c**). This could suggest that bacteria with low-affinity hydrogenases might be more active in the rhizosphere, as these bacteria are believed to grow on the high concentrations of H_2 produced in microniches, such as N_2-fixing root nodules (9), which are found in rhizosphere soil. The OTU representatives of the cDNA libraries stemming from the [NiFe]-244F/568R primer pair spanned numerous phylogenetic phyla based on RAxML EPA, such as *Actinobacteria*, *Proteobacteria*, *Verrucomicrobia*, *Acidobacteria*, *Planctomycetes* and *Chloroflexi* (**Fig. S10**).

Summary

Although similar patterns in diversity and community composition were observed across the investigated primer pairs (**Figs. 1, S4, S5**), the long-read primers targeting nearly the full-length of the group 1h [NiFe]-hydrogenase (*hhyL*) gene captured a wider breath of phylogenetic diversity in soil samples compared to previously published primers. These targeted investigations revealed putatively novel sequences in our nearly full-length group 1h [NiFe]-hydrogenase libraries. The average pairwise genetic distance among the nearly full-length sequences was ca. 50% generating ca. 2403 OTUs, compared to 18% or 25% from the shorter amplicon sequences generating 325 or 61 OTUs. Previous investigations noted a discrepancy between the theoretical and measured estimates of H_2-oxidizing bacteria in soil, and one of the discussed possible reasons were unidentified participants (5). Although it is unclear if we captured all of the group 1h [NiFe]-hydrogenase diversity in these soils, we submit that these newly developed primers capture more of the community, identifying some putative novel community members (**Fig. S6c**) for future investigations.

The goal of the cDNA libraries was to ascertain if acidobacterial group 1h [NiFe] hydrogenases were being expressed in the soil. No acidobacterial group 1h [NiFe]-hydrogenases were detected with [NiFe]-1129F/1640R primer pair, but were detected with our long-read primers (**Fig. 6c)** and in both the DNA- and cDNA-based libraries stemming from the primer pair [NiFe]-244F/568R (**Figs. S6a, S10**). Taken together this illustrates that the acidobacterial *hhyL* genes can be detected with the newly developed long-read primers and [NiFe]-244F/568R and appear to be expressed in the soil, as indicated in the cDNA-based libraries of the primer pair [NiFe]-244F/568R. Yet there was an increased genetic distance of acidobacterial/plancomycetes sequences amplified from the long-read primers (ca. 23%) compared to sequences stemming from [NiFe]-244F/568R (ca. 17%) indicating that this community was better captured with this new primer pair. This suggests that the additional sequence length,

primer location and/or degenerate nature of the primer pair are necessary to capture not only an increased acidobacterial group 1h [NiFe]-hydrogenase diversity, but also overall diversity in soils.

References

1. Constant P, Chowdhury SP, Pratscher J, Conrad R (2010) Streptomycetes contributing to atmospheric molecular hydrogen soil uptake are widespread and encode a putative high-affinity [NiFe]-hydrogenase. *Environ Microbiol* 12:821–829.

2. Constant P, Chowdhury SP, Hesse L, Conrad R (2011) Co-localization of atmospheric H_2 oxidation activity and high affinity H_2-oxidizing bacteria in non-axenic soil and sterile soil amended with *Streptomyces* sp. PCB7. *Soil Biol Biochem* 43:1888–1893.

3. Angel R (2012) Total Nucleic Acid Extraction from Soil. *Protocol Exchange*. doi:10.1038/protex.2012.046.

4. Herbold CW, *et al*. (2015) A flexible and economical barcoding approach for highly multiplexed amplicon sequencing of diverse target genes. *Front Microbiol* 6:731.

5. Constant P, Chowdhury SP, Hesse L, Pratscher J, Conrad R (2011) Genome data mining and soil survey for the novel group 5 [NiFe]-hydrogenase to explore the diversity and ecological importance of presumptive high-affinity H_2-oxidizing bacteria. *Appl Environ Microbiol* 77:6027–6035.

6. Jain C, Rodriguez-R LM, Phillippy AM, Konstantinidis KT, Aluru S (2018) High throughput ANI analysis of 90K prokaryotic genomes reveals clear species boundaries. *Nat Com* 9:5114.

7. Piché-Choquette S, Khdhiri M, Constant P (2017) Survey of high-affinity H_2-oxidizing bacteria in soil reveals their vast diversity yet underrepresentation in genomic databases. *Microb Ecol* 74:771-775.

8. Søndergaard D, Pedersen CNS, Greening C (2016) HydDB: A web tool for hydrogenase classification and analysis. *Sci Reports*. 6:1–8.

9. Maimaiti J, Zhang Y, Yang J, Cen YP, Layzell DB, Peoples M, *et al*. (2007) Isolation and characterization of hydrogen-oxidizing bacteria induced following exposure of soil to hydrogen gas and their impact on plant growth. *Environ Microbiol*. 9:435–444.

SI -2. Investigating the *hhyS* homolog in KBS 83 and *E. aggregans*

A homologous gene to *hhyS* was detected upstream (ca. 3600 bp) of the structural and maturation genes of the group 1h [NiFe]-hydrogenase in *Acidobacteriaceae* bacterium KBS 83 (G002DRAFT_03432) and *E. aggregans* (Q363DRAFT_01016). These homologues were distantly related to the *hhyS* gene in each respective genome (ca. 70.7% amino acid sequence similarity based on ca. 320 amino acids across the strains). In comparison, the *hhyS* of *Acidobacteriaceae* bacterium

KBS 83 and *E. aggregans* had an amino acid sequence similarity of 87.7% (based on ca. 320 amino acids). As seen with *P. methylaliphatogenes*, the homolog appears to be a part of a putative two-component sensory system (KBS 83: G002DRAFT_03432-03434; *E. aggregans*: Q363DRAFT_01014-01016).

The *hhyS* homolog in *Acidobacteriaceae* bacterium KBS 83 was expressed in both exponential (ca. 128 copies ng^{-1}) and stationary phase (ca. 2,153 copies ng^{-1}), with ca. 13-fold upregulation in stationary phase upon normalization to the 16S rRNA gene in KBS 83. The *hhyS* homolog was not expressed in neither exponential nor stationary phase for *E. aggregans*.

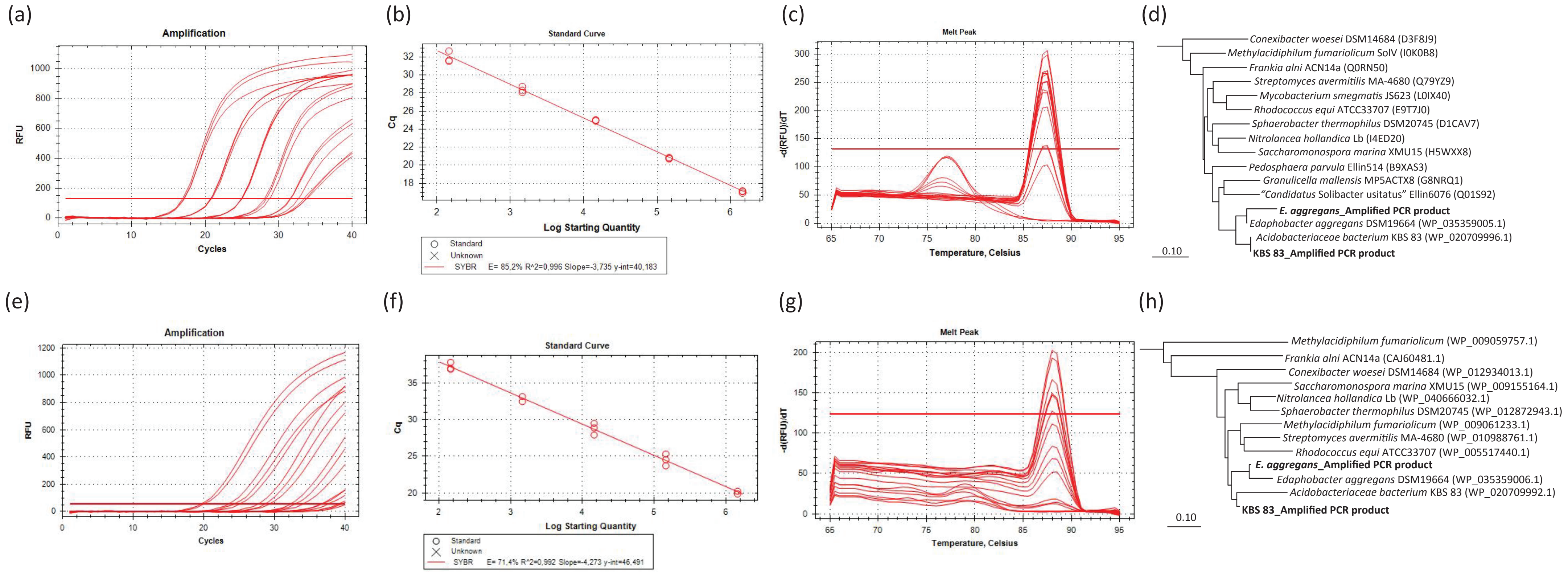

Figure S1. Establishment of the acidobacterial strain-specific group 1h [NiFe]-hydrogenase *hhyL* (**panels a-d**) and *hhyS* (**panels e-h**) qPCR assays. qPCR amplification (**panels a,e**), standards (**panels b,f**), melting curves (**panels c,g**) are depicted for a serially diluted DNA standard. Neighbor-joining phylogenetic tree of amplified products illustrating specificity of the reaction (**panels d, h**). Data from *Acidobacteriaceae* bacterium KBS 83 strain specific primers are shown for qPCR amplification, standards and melting curves. Similar patterns were observed for *E. aggregans*.

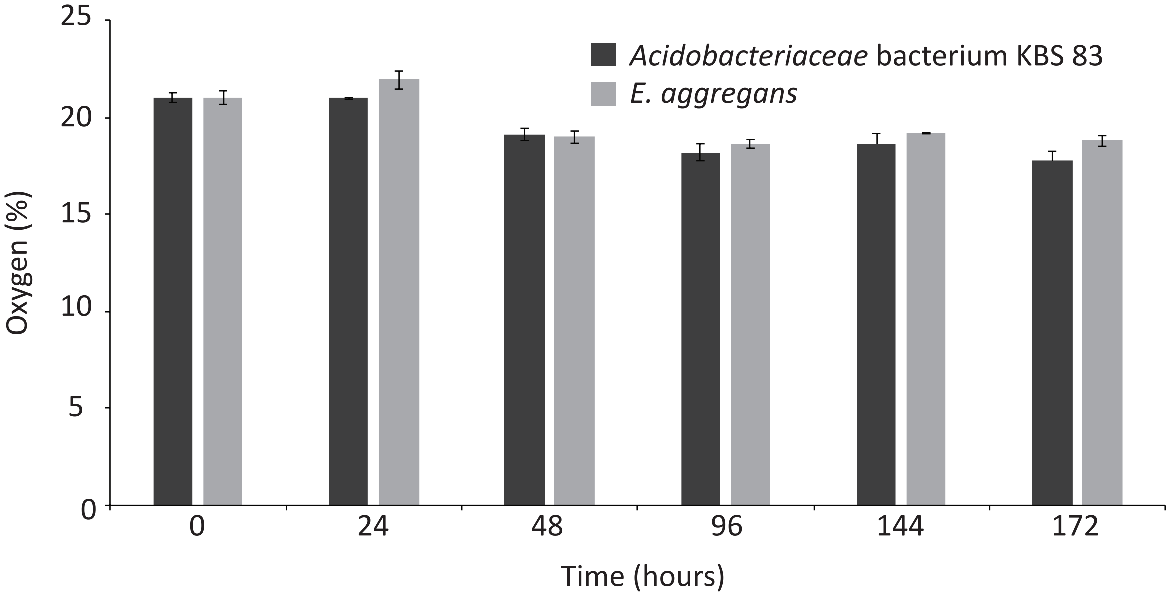

Figure S2. Oyxgen concentration in the headspace of *Acidobacteriaceae* bacterium KBS 83 & *E. aggregans* cultures during H_2consumption assay.

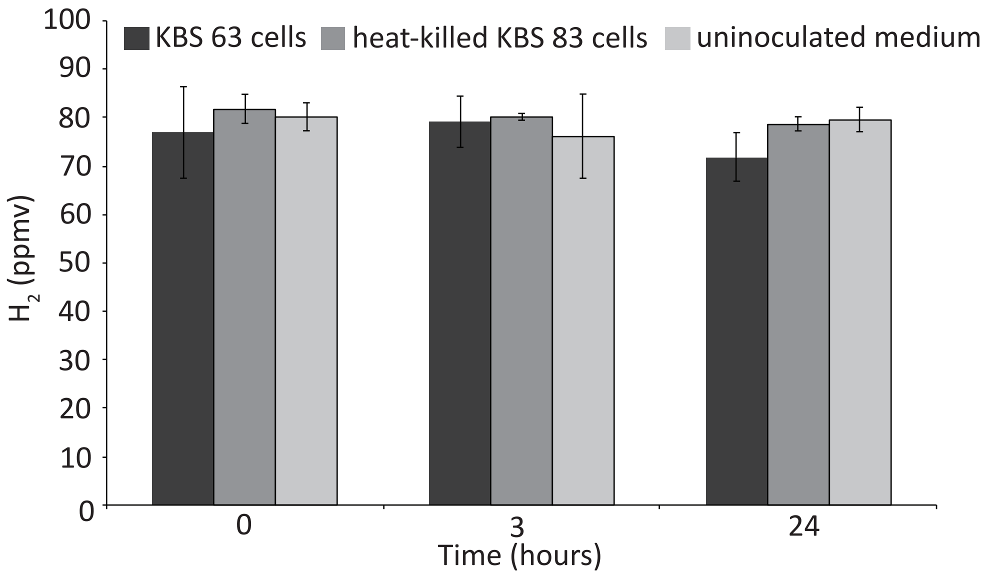

Figure S3. H_2 consumption controls over 24 hours for *T. roseus* KBS 63 (group 1h [NiFe]-hydrogenase negative strain) and *Acidobacteriaceae* bacterium KBS 83 (group 1h [NiFe]-hydrogenase positive strain), along with uninoculated medium. No significant difference in H_2 concentrations was found among the three controls over time ($P>0.73$).

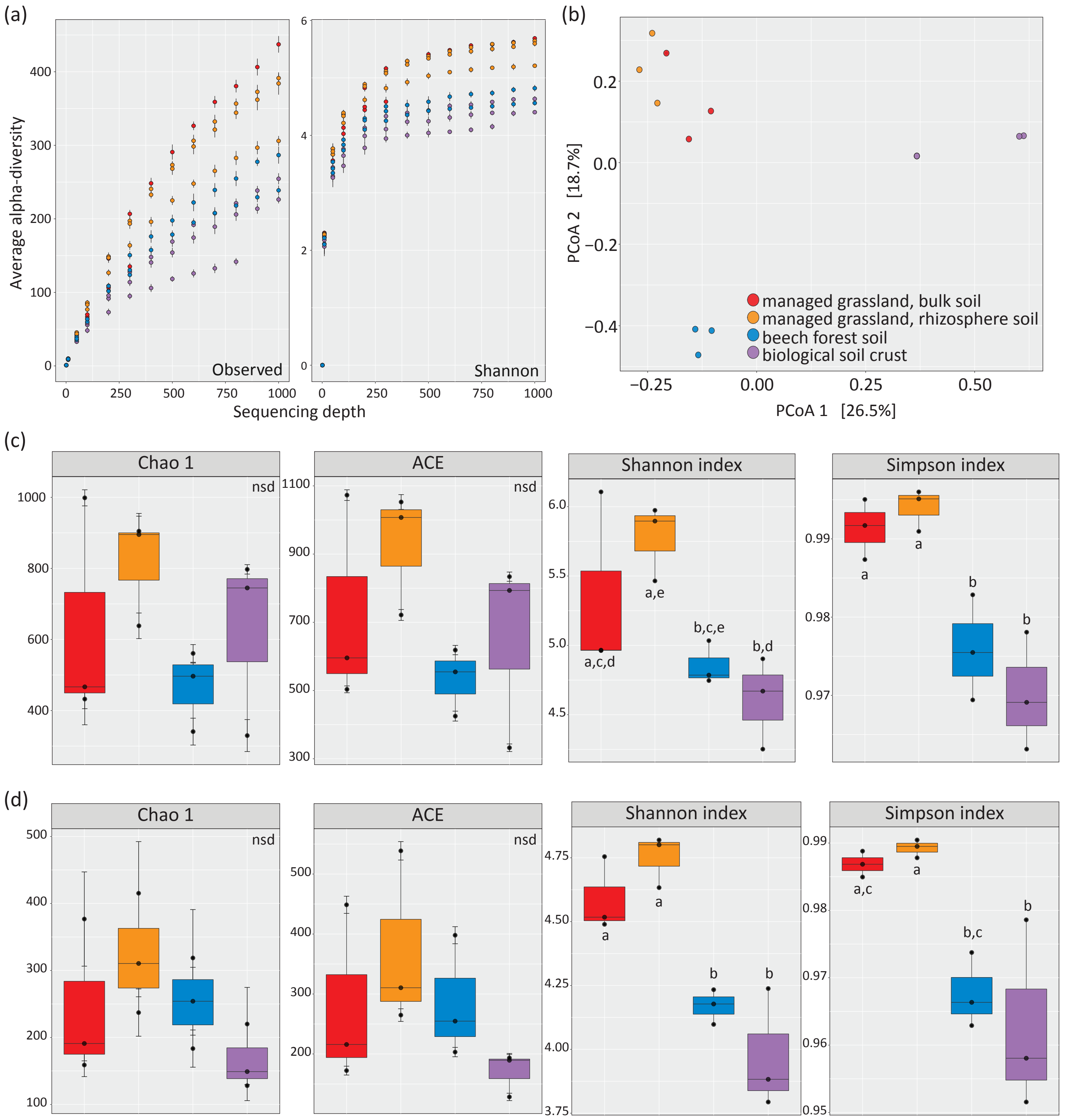

Figure S4. Diversity statistics for the long-read libraries of the group 1h [NiFe]-hydrogenase across the four soil types: managed grassland soil (red), rhizosphere soil (orange), beech forest soil (blue) and biological soil crust (purple). Rarefaction analysis based on observed and Shannon index (**panel a**); beta diversity (Bray-Curtis dissimilarity) of unrarefied data (**panel b**) and diversity statistics of unrarefied (**panel c**) and rarefied data (**panel d**). Data were unrarefied for **panels b & c**, but rarefied in **panel d**. Analysis of variance with a Tukey's HSD mean separation was performed across the soil types for the diversity statistics for the unrarefied and rarefied data; similar letters indicate that no significant difference was observed ($P > 0.05$), 'nsd' = no significant difference. Legend for all panels can be found in **panel c**.

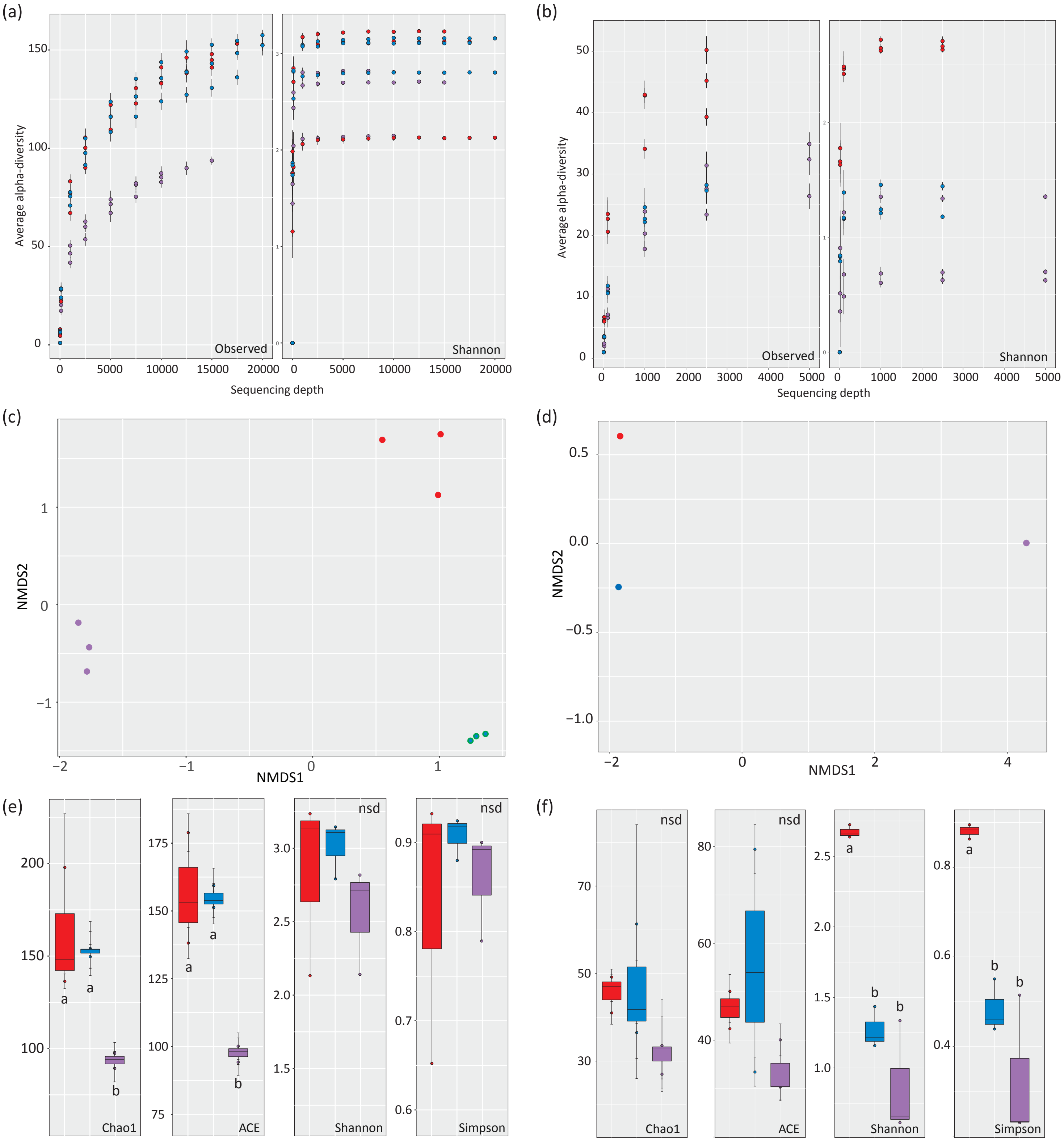

Figure S5. Diversity statistics for the short-read fragment libraries of the *hhyL* gene for three soil types: managed grassland soil (red), beech forest soil (blue) and biological soil crust (purple). Rarefaction analysis for [NiFe]-244F/568R (**panel a**) and [NiFe]-1129F/1640R (**panel b**); NMDS based on Bray-Curtis for [NiFe]--244F/568R (**panel c**) and [NiFe]-1129F/1640R (**panel d**); and diversity statistics for [NiFe]-244F/568R (**panel e**) and [NiFe]-1129F/1640R (**panel f**). Note - triplicate libraries stemming from [NiFe]-1129F/1640R are depicted in **panel d**, but the libraries from each soil type cluster very closely. Analysis of variance with a Tukey's HSD mean separation was performed across the soil types for the diversity statistics; similar letters indicate that no significant difference was obserbedf (P > 0.05), 'nsd' = no significant difference. Libraries stemming from [NiFe]244F/568R were rarified to 10,000 sequences & from [NiFe]-1129f/1640R to 2,000 sequences.

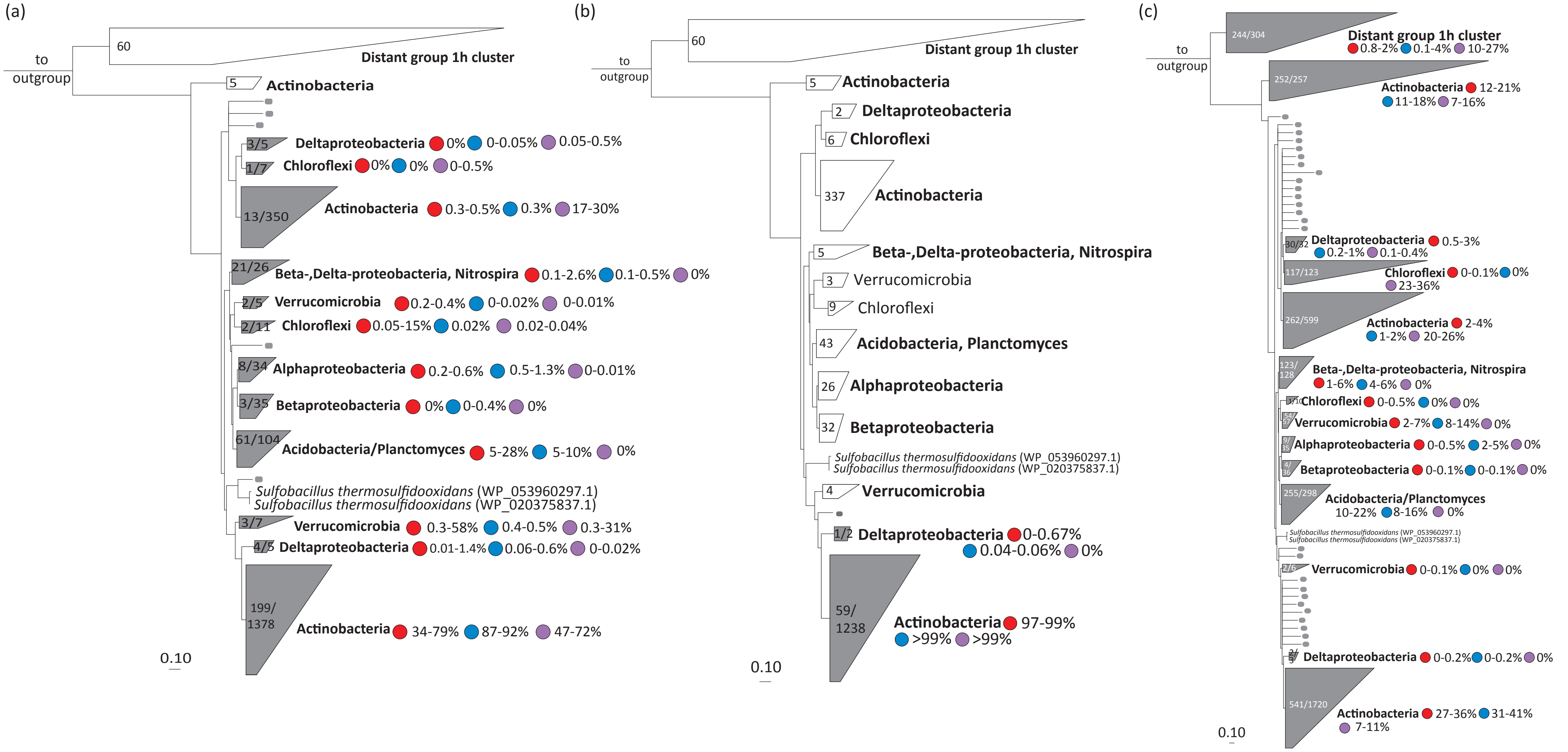

Figure S6. RAxML-EPA tree of the group 1h [NiFe]-hydrogenase *hhyL* based on amino acid sequences. The phylogenetic placements of OTU representatives (in gray collapsed clusters) are shown stemming from the [NiFe]-244F/568R (**panel a**), [NiFe]-1129F/1640R primer pairs (**panel b**) and the newly designed long-read primer pair (**panel c -** same as Figure 2b, here for comparison of primer pair sets). The number of placed short reads group 1h [NiFe]-hydrogenase and total number of sequences in each cluster are depicted. The proportion of sequences within each soil type are depicted to the right of the taxonomic groups: managed grassland (red), beech forest (blue), and biological soil crust (purple). Sequences from group 1g [NiFe]-hydrogenases were used as an outgroup. The scale bar indicates the number of substitutions per site.

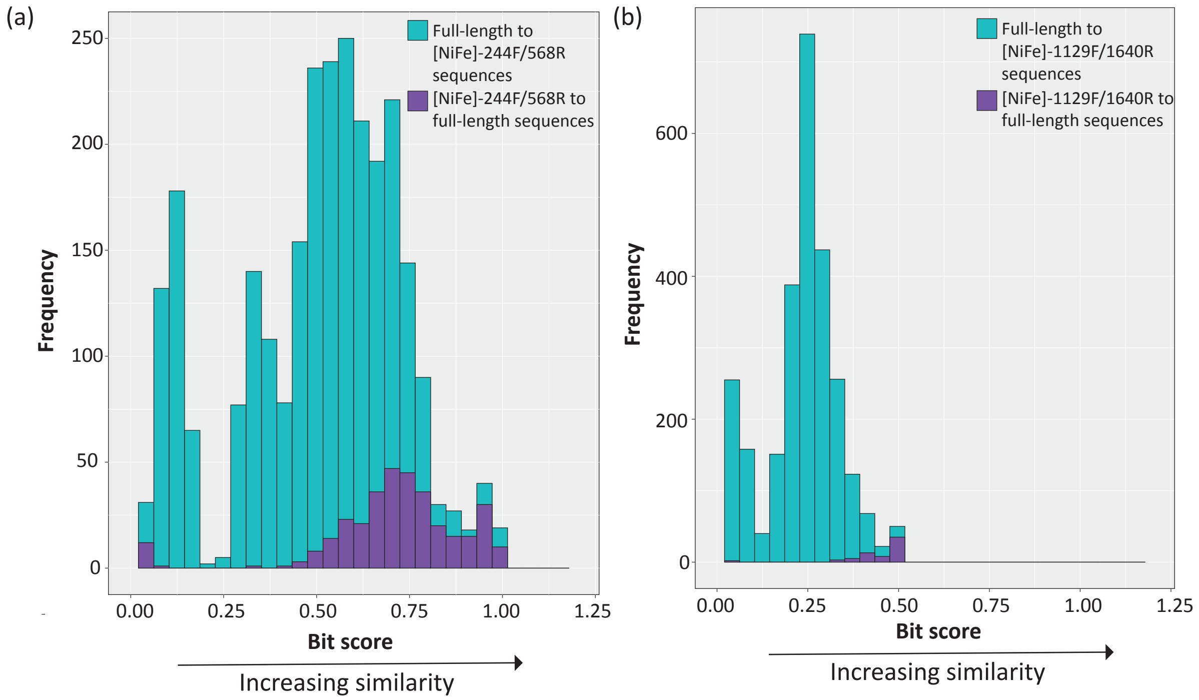

Figure S7. Assessing the overlap of the group 1h [NiFe]-hydrogenase communities using bidirectional nucleotide blast analyses: [NiFe]-244F/568R to nearly full-length sequences (**panel a**) and [NiFe]-1129F/1640R to nearly full-length sequenes (**panel b**). Top bit scores were normalized to self-nucleotide blast of either [NiFe]-244F/568R (**panel a**) or [NiFe]-1129F/1640R (**panel b**).

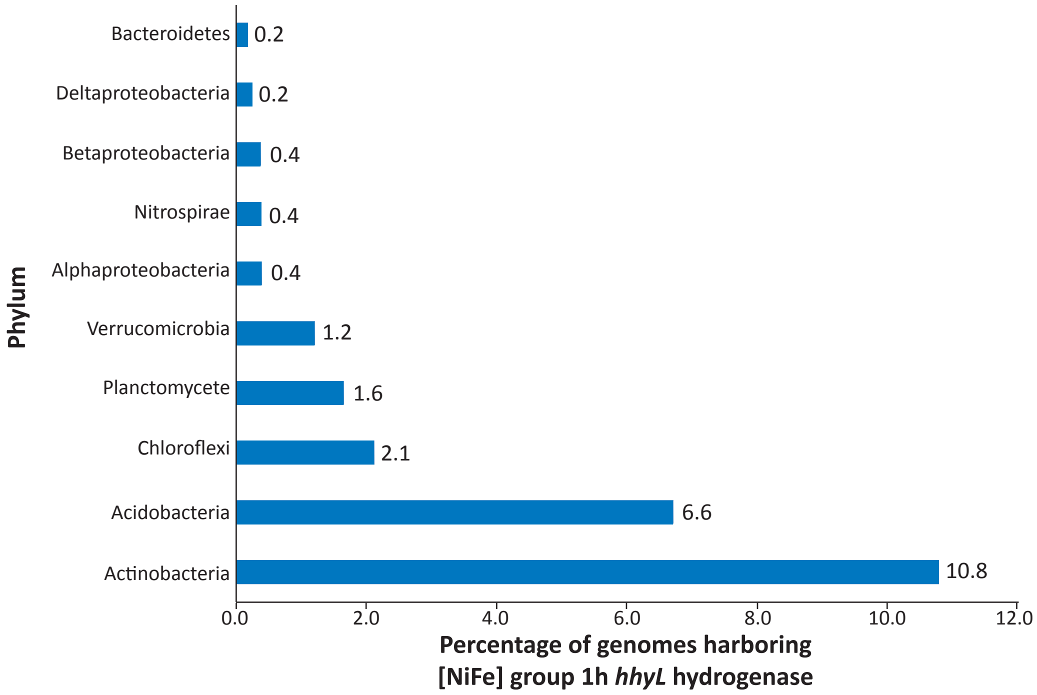

Figure S8. Percentage of genomes and metagenome assembled genomes (MAGs) harboring the group 1h [NiFe]-hydrogenase gene (*hhyL*) across various bacterial phyla. Publicly available genomes and MAGs were screened for this hydrogenase using an hmm model.

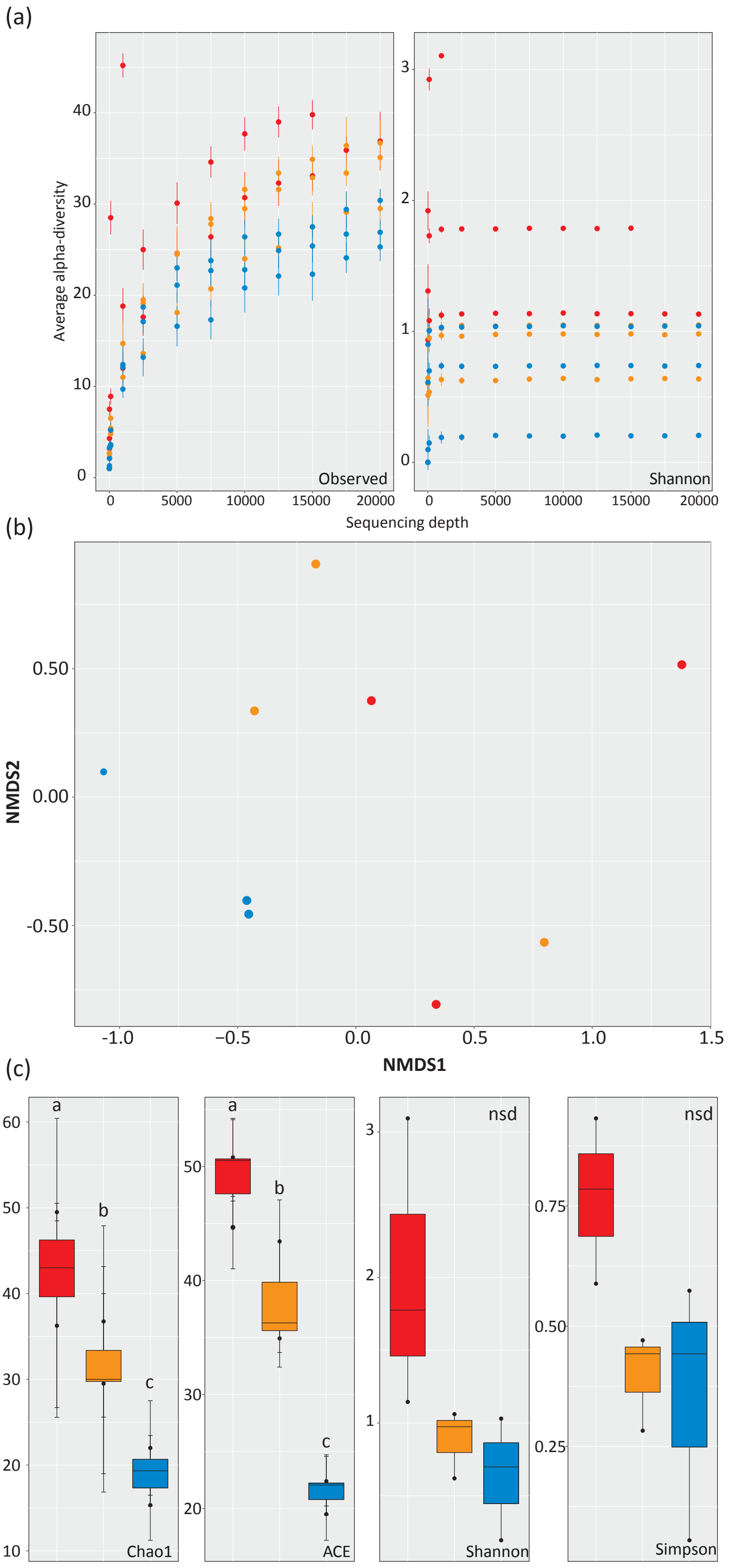

Figure S9. Diversity statistics of the *hhyL* sequences amplified from cDNA with primer pair ([NiFe]-244F/568R) for three soil types: managed grassland soil (red), rhizosphere soil (orange) and beech forest soil (blue). Rarefaction analysis (**panel a**); NMDS based on Bray--Curtis (**panel b**) and diversity statistics (**panel c**). Analysis of variance with a Tukey's HSD mean separation was performed across the soil types for the diversity statistics; similar letters indicate that no significant difference was observed (P > 0.05), 'nsd' = no significant difference. Libraries were rarified to 2,300 sequences.

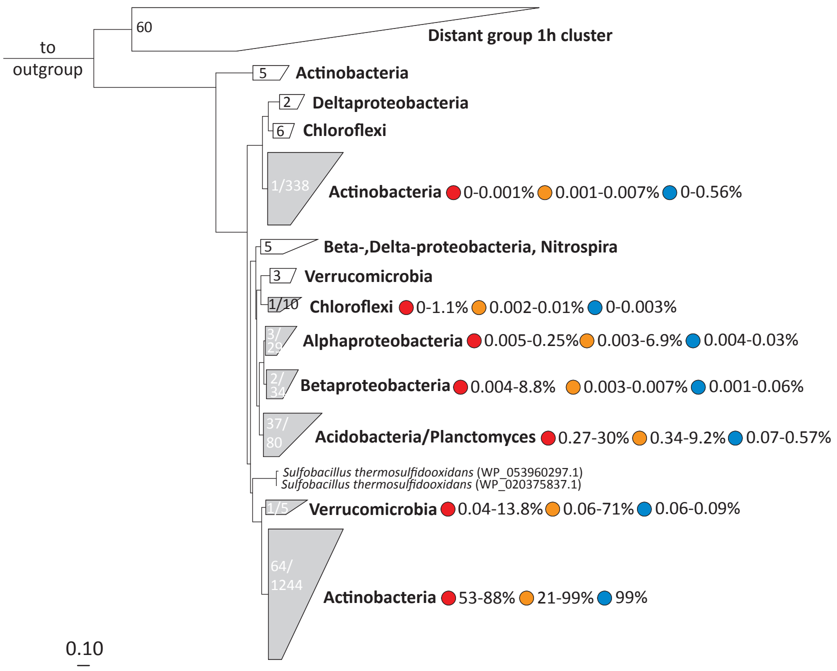

Figure S10. RAxML-EPA tree of the group 1h [NiFe]-hydrogenase (*hhyL*) based on amino acid sequences. The phylogenetic placements of OTU representatives (in gray collapsed clusters) from cDNA amplified with primer pair ([NiFe]-244F/568R) are shown. The number of placed short read group 1 [NiFe]-hydrogenase gene (*hhyL*) sequences and total number of sequences in each cluster are depicted. The proportion of sequences within each soil are depicted to the right of the taxonomic groups: managed grassland (red), rhizosphere (orange) and beech forest (blue). The outgroup was *Thermoproteus neutrophilus* (WP_012349775.1) and *Pyrobaculum islandium* (group 1g; WP_011761956.1). Scale bar indicates the number of substitu tions per site.

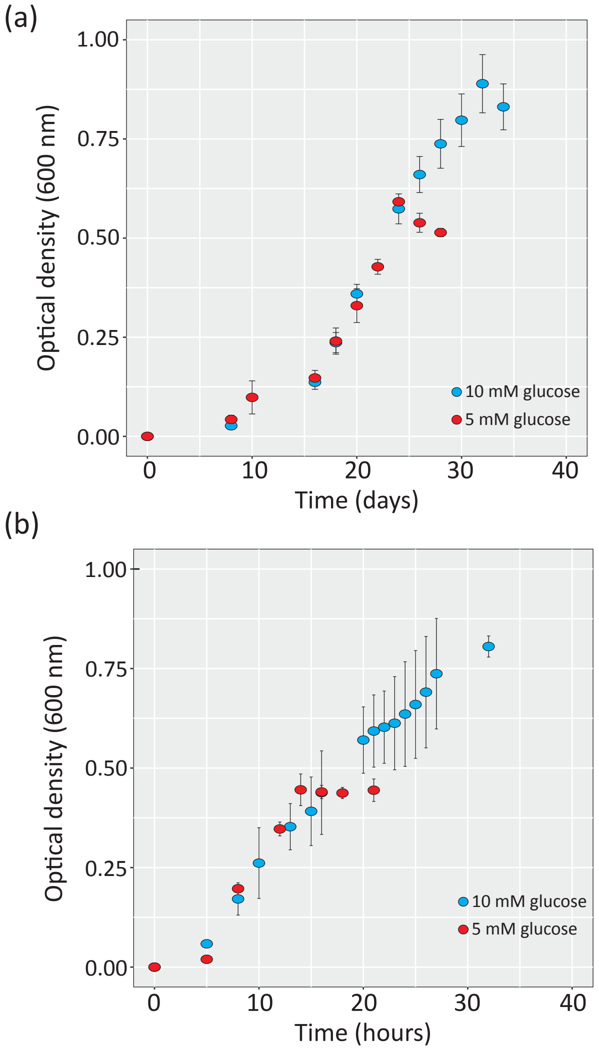

Figure S11. Defining carbon-limiting conditions across the investigated acidobacterial strains, *Acidobacteriaceae* bacterium KBS 83 (**panel a**) and *E. aggregans* (**panel b**). To determine carbon-limiting conditions, strains were grown in the VSB-6 defined medium (see Materials and Methods for more details on the medium composition) in differing glucose concentrations (5 mM and 10 mM glucose). Carbon-limiting conditions were defined when the cellular yield (optical density 600 nm) was proportional to the amount of carbon provided. Growth was monitored by measuring the optical density at 600 nm.

(a)

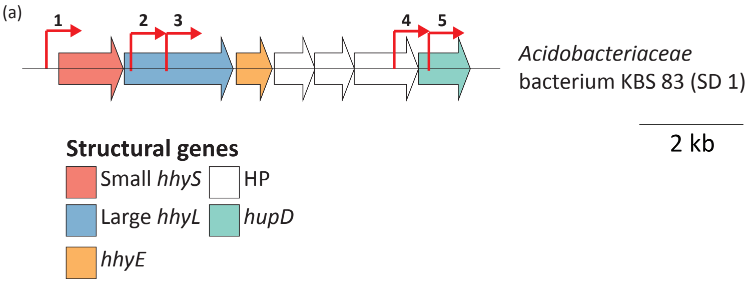

(b)

No.	Arbitrary position	Score	Transcrip. Facotrs	Score
1	710	0.831	rpoD15<br>nagC	694<br>724
2	2087	0.688	argR2<br>crp	2032<br>2050
3	2752	0.339	ND	ND
4	5504	0.820	ND	ND
5	5895	0.938	ND	ND

Figure S12. Identification of predicted promoter regions (depicted with red arrows) across the structural genes for the group 1h [NiFe]-hydrogenase operon in strain *Acidobacteriaceae* bacterium KBS 83 (**panel a**). Predicted promoter position and transcription factors, along with the respective scores (**panel b**). Data were generated using convolutional neural network models (https://arxiv.org/abs/1610.00121).

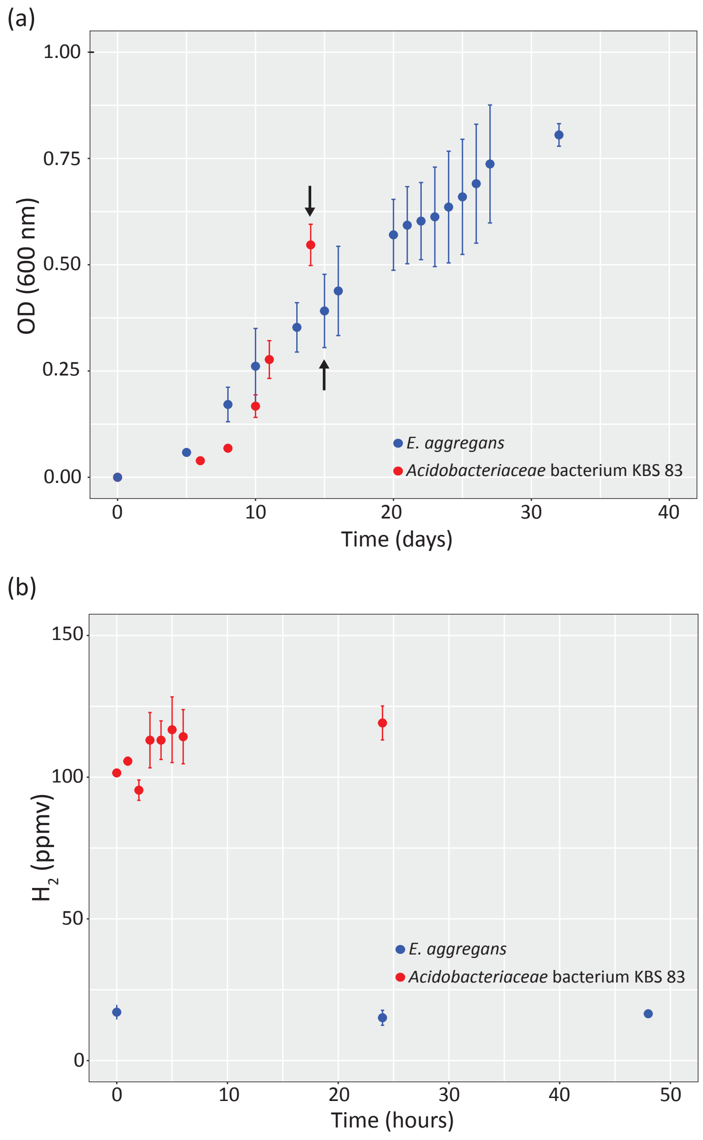

Figure S13. H_2 consumption assays of cells in exponential phase. Cells were harvested for both strains in exponential phase (**panel a**); arrows depict the time points in which the cells were harvested from each respective strain. Controls for this assay can be found in Figure S3. H_2consumption of the exponential phase cells of both strains was monitored over time (**panel b**). No significant H_2 consumption was observed over time for neither *E. aggregans* (P > 0.71), nor *Acidobacteriaceae* bacterium KBS 83 (P > 0.064).

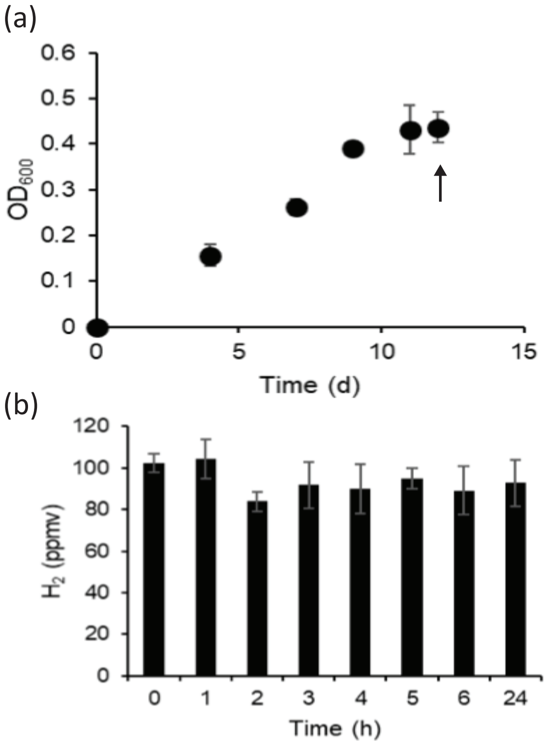

Figure S14. Growth curve of *E. aggregans* grown on an undefined medium (R2A medium, without agar) under aerobic conditions (**panel a**). Arrow depicts time point where H_2 consumption was measured over a 24-hour period (**panel b**). No significant H_2 consumption was detected over a 24-hour period ($P > 0.18$). The controls for this experiment can be found in Figure S3.

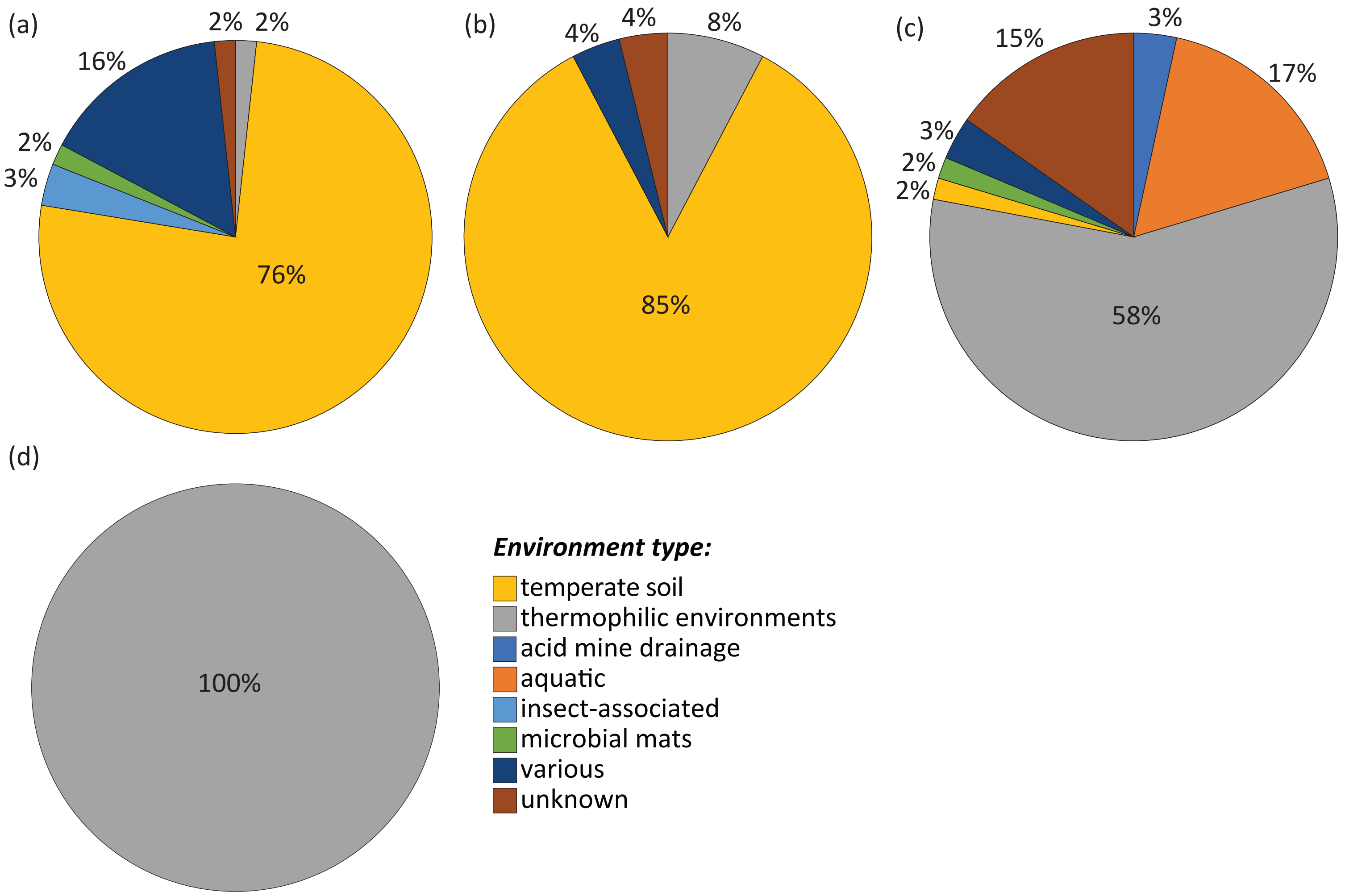

Figure S15. Distribution of the (**a**) *Edaphobacter aggregans* (group 1h [NiFe]-hydrogenase containing strain) (**b**) *Acidobacteriaceae* bacterium KBS 83 (group 1h [NiFe]-hydrogenase containing strain), (**c**) *Acidobacterium ailaaui* (group 1f [NiFe]-hydrogenase containing strain) and (**d**) *Pyrinomonas methylaliphatogenes* K22 (group 1h [NiFe]-hydrogenase containing strain) in the environment. Full-length 16S rRNA gene sequences for each respective strain (*E. aggregans*, DQ528761.1, 1,425 bp; *Acidobacteriaceae* bacterium KBS 83, FJ870383.1, 1,418 bp; *A. ailaaui*, KX306477.1, 1,258 bp; *Pyrinomonas methylaliphatogenes* K22, AM749787.1, 1,432bp) were blasted in NCBI using Megablast; a total of 59 (*Acidobacterium ailaaui*), 58 (*Edaphobacter aggregans)*, 26 (*Acidobacteriaceae* bacterium KBS 83) and 2 (*Pyrinomonas methylaliphatogenes* K22) sequences were recovered from the public database (NCBI) having a similiarity of $\geq$97%. These were classified manually as orginating from "temperate soils", "thermophilic environments (such as soils, bagasse and volcanic deposits, all with temperatures >40˚C), "acid mine drainage", "aquatic" (such as deep-sea octacoral samples), "insect-associated" (such as the termite hindgut and gut of New Zealand's endemic Huhubeetle larvae), "microbial mats" "various" (such as "hot water", "leaves", "skin" and "waste pile") and "unknown" (no description was given in the sequence entries at NCBI, along with no associated manuscript). The percent of these sequences found in each respective environment is depicted.

Table S1. Primers used in this study.

**Name**	**Target gene**	**Sequence (5' to 3')**	**Reference**
Grp1h_Full_F	[NiFe] group 1h LSU hydrogenase	TTA CCT CCC GTA TTT GCG GNR TNT GYG G	This study
Grp1h_Full_R		CCG CAC GGC AGG CAN GGR TCR AA	This study
KBS83_grp1h_LSU_F	Strain specific (KBS 83) LSU [NiFe] group 1h hydrogenase	TAC ACC TAT CAG AAG ATC ACC	This study
KBS83_ grp1h_LSU_R		CGC CCG TGT CGA GGG CGA GGT	This study
Eagg_ grp1h_LSU_F	Strain specific (*E. aggregantus*) LSU [NiFe] group 1h hydrogenase	TAC ACG TAC GCG AAG ATG ACC	This study
Egg_ grp1h_LSU_R		CGC CCG TGT CGA GCG CGA GAT	This study
KBS83_ grp1h_SSU_F	Strain specific (KBS 83) SSU [NiFe] group 1h hydrogenase	TCC CAA ACG AGA GGA TCA AGA	This study
KBS83_ grp1h_SSU_R		GGA GAT AGA GGA CTG TCT CCA	This study
Eagg_ grp1h_SSU_F	Strain specific (*E. aggregantus*) SSU [NiFe] group 1h hydrogenase	TTC CAA ACG AGA AGA TCA AGC	This study
Egg_ grp1h_SSU_R		GCA GAT ACA GCA CCG TCT CCA	This study
KBS83_ grp1h_SSUhomolog_F	Strain specific (KBS 83) SSU homolog [NiFe] group 1h hydrogenase	ACG GGG ACA CCA TAG CAA TGA C	This study
KBS83_ grp1h_SSUhomolog_R		TTG GAA TCG AGC CCT CTA CGA C	This study
Eagg_ grp1h_SSUhomolog_F	Strain specific (*E. aggregantus*) SSU homolog [NiFe] group 1h hydrogenase	AGA TCG CAA CCT ATC GCT G	This study
Egg_ grp1h_SSUhomolog_R		TTC ACA AAC TCT TCC CCA TTC	This study
NiFe-244F	[NiFe] group 1h LSU hydrogenase	GGG ATC TGC GGG GAC AAC CA	(Constant, *et al.*, 2010)
NiFe- 568R	[NiFe] group 1h LSU hydrogenase	TCT CCC GGG TGT AGC GGC TC	(Constant, *et al.*, 2011)
NiFe-1129F	[NiFe] group 1h LSU hydrogenase	CCG CGG TGG TTC GAC GGC AA	(Constant et al., 2010)
NiFe-1640R	[NiFe] group 1h LSU hydrogenase	TGC ACG GCG TCC TCG TAC GG	(Constant et al., 2010)
Acid303Fa	16S rRNA gene	GCG CAC GGM CAC ACT GGA	(Steger, 2010)
Acid657R	16S rRNA gene	ATT CCA CKC ACC TCT CCC AYC AY	(Steger, 2010)

Constant, P., Chowdhury, S. P., Hesse, L., & Conrad, R. (2011). Co-localization of atmospheric H_2 oxidation activity and high affinity H_2-oxidizing bacteria in non-axenic soil and sterile soil amended with *Streptomyces sp*. PCB7. *Soil Biol.Biochem.*, *43*(9), 1888–1893. http://doi.org/10.1016/j.soilbio.2011.05.009

Constant, P., Chowdhury, S. P., Pratscher, J., & Conrad, R. (2010). Streptomycetes contributing to atmospheric molecular hydrogen soil uptake are widespread and encode a putative high-affinity [NiFe]-hydrogenase. *Environ. Microbiol.*, *12*(3), 821–829. http://doi.org/10.1111/j.1462-2920.2009.02130.x

Steger, D. (2010). Community structure and distribution of functional microbial groups within two complex environments. PhD Thesis. University of Vienna. http://doi.org/10.25365/thesis.10359

Table S2. Summary statistics of nearly full-length group 1h [NiFe]-hydrogenase sequences.

Soil	Barcode	Reads	OTUs at 95%	% OTUs unclassified (NCBI based)	% OTUs non-hydrogenase (HydDB based)
Beech forest soil _2012	pb1	1157	322	35%	0%
Beech forest soil _2013	pb10	390	190	33%	0%
Beech forest soil_2014	pb17	1770	435	42%	0%
Meadow grassland, bulk soil_r1	pb19	389	210	34%	0%
Meadow grassland, bulk soil_r2	pb21	3678	861	32%	0%
Meadow grassland, bulk soil_r3	pb29	211	160	30%	0%
Meadow grassland, rhizosphere soil_r1	pb32	1412	443	35%	0%
Meadow grassland, rhizosphere soil_r2	pb34	1353	578	30%	0%
Meadow grassland, rhizosphere soil_r3	pb35	1396	564	32%	0%
Biological soil crust_r1	pb38	9652	635	49%	0%
Biological soil crust_r2	pb40	818	179	51%	0%
Biological soil crust_r3	pb42	13849	739	51%	0%

Table S3. Number of sequences (% of total sequences) containing the respective primer region of the listed primers, along with different mismatch cases. Nearly-full length group 1h [NiFe]-hydrogenase sequences stemming from the PacBio sequencing run were used (n=2403; *except for region 1640R, where n=2242 sequences were used as there were sequences without sequence data at this region).

	**No. mismatches**				
	**0**	**1**	**2**	**3**	**>4**
NiFe-244F	3 (0.12%)	34 (1.41%)	266 (11.1%)	640 (26.6%)	1460 (60.8%)
NiFe-568R	4 (0.17%)	17 (0.71%)	108 (4.49%)	226 (9.40%)	2048 (85.2%)
NiFe-1129F	26 (1.08%)	165 (6.87%)	283 (11.8%)	172 (7.16%)	1757 (73.1%)
NiFe-1640R*	80 (3.57%)	299 (13.3%)	528 (23.6%)	461 (20.6%)	1035 (46.2%)

Table S4. Details on publicly available MAG seqeunces depicted in Figure 3.

Acidobacteria NiFe group 1h hydrogenases

							CheckM Analysis		
**Name in Fig. 3**	**genome**	**Environment**	**Reference**	**proteinID**	**Classification based on Genome Taxonomy Database**	**completeness**	**contamination**	**Heterogeneity**	**Subdivision**
AcidoMAG_soil HarvardForest	GCA_003751275.1_ASM375127v1	harvard forest soil	https://www.ncbi.nlm.nih.gov/protein/ROP69215.1/	ROP69215.1	d__Bacteria;p__Acidobacteriota;c__Acidobacteriae;o__Acidobacteriales;f__Acidobacteriaceae;g__KBS-89;s__	100	1.72	0	1
Acidisarcina polymorpha SBC82	GCA_003330725.1_ASM333072v1	soil	https://www.ncbi.nlm.nih.gov/pubmed/30510549	AXC10343.1	d__Bacteria;p__Acidobacteriota;c__Acidobacteriae;o__Acidobacteriales;f__Acidobacteriaceae;g__;s__	99.95	0	0	1
AcidoMAG_peatland soil Sweden	GCA_003134995.1_20111000_P2S	peat metagenome	https://www.ncbi.nlm.nih.gov/pubmed/30013118	PLDC01000024.1_25	d__Bacteria;p__Acidobacteriota;c__Acidobacteriae;o__Acidobacteriales;f__Koribacteraceae;g__Sulfotelmatobacter;s__Sulfotelmatobacter sp003134995	100	1.71	0	1
AcidoMAG_peatland soil Sweden	GCA_003169195.1_20110600_S1S	peat metagenome	https://www.ncbi.nlm.nih.gov/pubmed/30013118	PMQB01000436.1_44	d__Bacteria;p__Acidobacteriota;c__Acidobacteriae;o__Bryobacterales;f__Bryobacteraceae;g__Bog-105;s__Bog-105 sp003169195	90.97	0.43	0	3
AcidoMAG_peatland soil Sweden	GCA_003140295.1_20110800_P1M	peat metagenome	https://www.ncbi.nlm.nih.gov/pubmed/30013118	PLJR01000108.1_20	d__Bacteria;p__Acidobacteriota;c__Acidobacteriae;o__UBA7541;f__UBA7541;g__Palsa-189;s__Palsa-189 sp003140295	76.04	3.45	0	putative 2
AcidoMAG_peatland soil Sweden	GCA_003152355.1_20120700_P2M	peat metagenome	https://www.ncbi.nlm.nih.gov/pubmed/30013118	PMAA01000262.1_14	d__Bacteria;p__Acidobacteriota;c__Acidobacteriae;o__UBA7541;f__UBA7541;g__Palsa-147;s__Palsa-147 sp003152355	88	3.47	0	putative 2
AcidoMAG_peatland soil Sweden	GCA_003156775.1_20120600_P2S	peat metagenome	https://www.ncbi.nlm.nih.gov/pubmed/30013118	PMHS01000239.1_14	d__Bacteria;p__Acidobacteriota;c__Acidobacteriae;o__UBA7541;f__UBA7541;g__Palsa-295;s__Palsa-295 sp003153585	72.79	1.28	0	putative 3
AcidoMAG_peatland soil Sweden	GCA_003152195.1_20120700_P3D	peat metagenome	https://www.ncbi.nlm.nih.gov/pubmed/30013118	PLZN01000334.1_20	d__Bacteria;p__Acidobacteriota;c__Acidobacteriae;o__Acidobacteriales;f__Acidobacteriaceae;g__PALSA-350;s__PALSA-350 sp003152195	82.95	1.77	0	putative 2
AcidoMAG_peatland soil Sweden	GCA_003156785.1_20120600_P2M	peat metagenome	https://www.ncbi.nlm.nih.gov/pubmed/30013118	PMHT01000231.1_12	d__Bacteria;p__Acidobacteriota;c__Acidobacteriae;o__Acidobacteriales;f__Koribacteraceae;g__Bog-209;s__	80.62	6.58	0	1
AcidoMAG_peatland soil Sweden	GCA_003167075.1_20110700_P2M	peat metagenome	https://www.ncbi.nlm.nih.gov/pubmed/30013118	PMUD01000132.1_61	d__Bacteria;p__Acidobacteriota;c__Acidobacteriae;o__Acidobacteriales;f__Koribacteraceae;g__Sulfotelmatobacter;s__Sulfotelmatobacter sp003131635	83.45	0.85	0	1
AcidoMAG_MeadowSoil_AngeloCoast	GCA_003225435.1_ASM322543v1	soil	https://www.ncbi.nlm.nih.gov/pubmed/31110364	PYV88525.1	d__Bacteria;p__Acidobacteriota;c__Acidobacteriae;o__Acidobacteriales;f__Koribacteraceae;g__Gp1-AA142;s__Gp1-AA142 sp003224855	87.84	5.98	12.5	1
AcidoMAG_peatland soil Sweden	GCA_003136855.1_20111000_P1S	peat metagenome	https://www.ncbi.nlm.nih.gov/pubmed/30013118	PLDV01000039.1_25	d__Bacteria;p__Acidobacteriota;c__Acidobacteriae;o__Acidobacteriales;f__Koribacteraceae;g__Sulfotelmatobacter;s__Sulfotelmatobacter sp003134995	96.51	1.71	0	1
AcidoMAG_peatland soil Sweden	GCA_003161455.1_20110600_P3S	peat metagenome	https://www.ncbi.nlm.nih.gov/pubmed/30013118	PMOT01000104.1_16	d__Bacteria;p__Acidobacteriota;c__Acidobacteriae;o__Acidobacteriales;f__Koribacteraceae;g__Sulfotelmatobacter;s__Sulfotelmatobacter sp003134995	99.15	1.71	0	1
AcidoMAG_peatland soil Sweden	GCA_003167955.1_20110700_P3S	peat metagenome	https://www.ncbi.nlm.nih.gov/pubmed/30013118	PMUV01000030.1_25	d__Bacteria;p__Acidobacteriota;c__Acidobacteriae;o__Acidobacteriales;f__Koribacteraceae;g__Sulfotelmatobacter;s__Sulfotelmatobacter sp003134995	97.34	1.82	50	1
AcidoMAG_peatland soil Sweden	GCA_003156805.1_20120600_P2M	peat metagenome	https://www.ncbi.nlm.nih.gov/pubmed/30013118	PMHV01000061.1_72	d__Bacteria;p__Acidobacteriota;c__Acidobacteriae;o__Acidobacteriales;f__Koribacteraceae;g__Sulfotelmatobacter;s__Sulfotelmatobacter sp003156805	95.77	2.56	0	1
AcidoMAG_peatland soil Sweden	GCA_003156615.1_20120600_P3M	peat metagenome	https://www.ncbi.nlm.nih.gov/pubmed/30013118	PMHI01000675.1_15	d__Bacteria;p__Acidobacteriota;c__Acidobacteriae;o__Acidobacteriales;f__Koribacteraceae;g__Sulfotelmatobacter;s__	88.46	4.44	16.67	1
AcidoMAG_peatland soil Sweden	GCA_003158215.1_20120600_P1M	peat metagenome	https://www.ncbi.nlm.nih.gov/pubmed/30013118	PMIA01000256.1_10	d__Bacteria;p__Acidobacteriota;c__Acidobacteriae;o__UBA7541;f__UBA7541;g__Palsa-295;s__Palsa-295 sp003153585	70.35	2.18	0	putative 3
AcidoMAG_peatland soil Sweden	GCA_003153585.1_20120700_P2S	peat metagenome	https://www.ncbi.nlm.nih.gov/pubmed/30013118	PLZZ01000189.1_18	d__Bacteria;p__Acidobacteriota;c__Acidobacteriae;o__UBA7541;f__UBA7541;g__Palsa-295;s__Palsa-295 sp003153585	78.91	1.71	0	putative 3
AcidoMAG_peatland soil Sweden	GCA_003166795.1_20110700_P3M	peat metagenome	https://www.ncbi.nlm.nih.gov/pubmed/30013118	PMUS01000010.1_5	d__Bacteria;p__Acidobacteriota;c__Acidobacteriae;o__UBA7541;f__UBA7541;g__Palsa-295;s__Palsa-295 sp003166795	93.45	5.7	0	putative 3
AcidoMAG_peatland soil Sweden	GCA_003132485.1_20120500_P23	peat metagenome	https://www.ncbi.nlm.nih.gov/pubmed/30013118	PKXX01000154.1_1	d__Bacteria;p__Acidobacteriota;c__Acidobacteriae;o__UBA7541;f__UBA7541;g__Palsa-295;s__Palsa-295 sp003166795	82.9	4.27	0	putative 3
AcidoMAG_peatland soil Sweden	GCA_003166995.1_20110700_P2S	peat metagenome	https://www.ncbi.nlm.nih.gov/pubmed/30013118	PMUH01000138.1_18	d__Bacteria;p__Acidobacteriota;c__Acidobacteriae;o__UBA7541;f__UBA7541;g__Palsa-295;s__Palsa-295 sp003131985	71.34	3.42	50	putative 3
AcidoMAG_peatland soil Sweden	GCA_003131635.1_20120500_P32	peat metagenome	https://www.ncbi.nlm.nih.gov/pubmed/30013118	PKVZ01000266.1_8	d__Bacteria;p__Acidobacteriota;c__Acidobacteriae;o__Acidobacteriales;f__Koribacteraceae;g__Sulfotelmatobacter;s__Sulfotelmatobacter sp003131635	90.05	3.42	50	1
AcidoMAG_peatland soil Sweden	GCA_003162515.1_20110600_P1M	peat metagenome	https://www.ncbi.nlm.nih.gov/pubmed/30013118	PMOD01000102.1_23	d__Bacteria;p__Acidobacteriota;c__Acidobacteriae;o__Acidobacteriales;f__Koribacteraceae;g__Sulfotelmatobacter;s__Sulfotelmatobacter sp003131635	74.89	0.85	0	1
AcidoMAG_peatland soil Sweden	GCA_003139395.1_20110800_P3S	peat metagenome	https://www.ncbi.nlm.nih.gov/pubmed/30013118	PLIS01000326.1_13	d__Bacteria;p__Acidobacteriota;c__Acidobacteriae;o__Acidobacteriales;f__Koribacteraceae;g__Sulfotelmatobacter;s__	87.76	8.55	45.45	1
AcidoMAG_peatland soil Sweden	GCA_003224855.1_ASM322485v1	peat metagenome	https://www.ncbi.nlm.nih.gov/pubmed/30013118	PYV57583.1	d__Bacteria;p__Acidobacteriota;c__Acidobacteriae;o__Acidobacteriales;f__Koribacteraceae;g__Gp1-AA142;s__Gp1-AA142 sp003224855	85.8	1.76	0	1
AcidoMAG_peatland soil Sweden	GCA_003166775.1_20110700_P3M	peat metagenome	https://www.ncbi.nlm.nih.gov/pubmed/30013118	PMUT01000141.1_7	d__Bacteria;p__Acidobacteriota;c__Acidobacteriae;o__UBA7541;f__UBA7541;g__Palsa-147;s__Palsa-147 sp003152355	85.92	2.56	66.67	putative 2
AcidoMAG_peatland soil Sweden	GCA_003141135.1_20120800_P3M	peat metagenome	https://www.ncbi.nlm.nih.gov/pubmed/30013118	PLKO01000300.1_6	d__Bacteria;p__Acidobacteriota;c__Acidobacteriae;o__UBA7541;f__UBA7541;g__Palsa-147;s__Palsa-147 sp003152355	79.24	5.13	0	putative 2
AcidoMAG_peatland soil Sweden	GCA_003132465.1_20120500_P23	peat metagenome	https://www.ncbi.nlm.nih.gov/pubmed/30013118	PKXW01000166.1_4	d__Bacteria;p__Acidobacteriota;c__Acidobacteriae;o__UBA7541;f__UBA7541;g__Palsa-147;s__Palsa-147 sp003152355	81.51	3.42	25	putative 2
AcidoMAG_peatland soil Sweden	GCA_003136675.1_20110800_S3S	peat metagenome	https://www.ncbi.nlm.nih.gov/pubmed/30013118	**PLFE01000211.1**_80	d__Bacteria;p__Acidobacteriota;c__Acidobacteriae;o__Bryobacterales;f__Bryobacteraceae;g__Bog-113;s__Bog-113 sp003136675	91.67	0.88	0	3

Planctomycetes NiFe group 1h hydrogenases

							CheckM Analysis	
**Name in Fig. 3**	**genome**	**Environment**	**Reference**	**proteinID**	**Classification based on Genome Taxonomy Database**	**completeness**	**contamination**	**Heterogeneity**
PlanctoMAG_unk.env(DHJA01000227.1)	GCA_002404095.1_ASM240409v1	unknown	https://www.ncbi.nlm.nih.gov/pubmed/28894102	DHJA01000227.1_28	d__Bacteria;p__Planctomycetota;c__Planctomycetes;o__Gemmatales;f__Gemmataceae;g__UBA4732;s__UBA4732 sp002404095	91.16	3.49	25
PlanctoMAG_aquatic env.(HBI46143.1)	GCA_003508275.1_ASM350827v1	aquatic	https://www.ncbi.nlm.nih.gov/pubmed/22740668	HBI46143.1	d__Bacteria;p__Planctomycetota;c__Planctomycetes;o__Gemmatales;f__Gemmataceae;g__UBA4732;s__UBA4732 sp002404095	83.7	2.33	33.33
PlanctoMAG_unk.env(SIN71083.1)	GCA_900129635.1_IMG-taxon_26954	harvard forest soil	https://www.ncbi.nlm.nih.gov/bioproject/?term=PRJEB18215	SIN71083.1	d__Bacteria;p__Planctomycetota;c__Planctomycetes;o__Isosphaerales;f__Isosphaeraceae;g__Singulisphaera;s__Singulisphaera sp900129635	99.61	5.81	0
PlanctoMAG_aquatic env. (JH621483.1)	GCA_000255675.1_ASM25567v1	aquatic	https://www.ncbi.nlm.nih.gov/pubmed/22740668	JH621483.1_121	d__Bacteria;p__Planctomycetota;c__Planctomycetes;o__Isosphaerales;f__Isosphaeraceae;g__Singulisphaera;s__Singulisphaera acidiphila	98.45	6.98	0

Other NiFe group 1h hydrogenases

							CheckM Analysis	
**Name in Fig. 3**	**genome**	**Environment**	**Reference**	**proteinID**	**Classification based on Genome Taxonomy Database**	**completeness**	**contamination**	**Heterogeneity**
BetaproteoMAG	GCA_003450855.1_ASM345085v1	unknown	https://www.ncbi.nlm.nih.gov/pubmed/30148503	HAG76002.1	d__Bacteria;p__Proteobacteria;c__Gammaproteobacteria;o__Burkholderiales;f__Rhodocyclaceae;g__Thauera;s__Thauera sp000310145	80.62	2.32	44.44
NitrospiraMAG	GCA_002083365.1_ASM208336v1	drinking water systm	https://www.ncbi.nlm.nih.gov/pubmed/28390307	OQW30533.1	d__Bacteria;p__Nitrospirota;c__Nitrospiria;o__Nitrospirales;f__Nitrospiraceae;g__Nitrospira;s__Nitrospira sp002083365	95.85	3.69	20

Table S5. Determining apparent kinetic parameters (average ± standard error) for H_2 oxidization on whole cells for strain *Acidobacteriaceae* bacterium KBS 83 and *E. aggregans* using two methods (nonlinear regression and Hanes-Woolf plot).

	Acidobacteriaceae bacterium KBS 83		*E. aggregans*	
	$V_{max\ app}$[a]	$K_{m\ app}$[c]	$V_{max\ app}$[b]	$K_{m\ app}$[c]
Nonlinear regression	0.27 ± 0.03	173 ± 63	6.16 ± 1.16	95 ± 31
Hanes-Woolf plot	0.21 ± 0.03	110 ± 57.9	6.20 ± 1.43	103 ± 19.0
Average	0.24	137	6.18	99

[a]Rates given as μmols mg^{-1} protein h^{-1}
[b]Rates given as nmols mg^{-1} protein h^{-1}
[c]Concentrations given in nM
